# Systematic identification and comparison of expressed profiles of lncRNAs and circRNAs with associated co-expression and ceRNA networks in mouse germline stem cells

**DOI:** 10.18632/oncotarget.15719

**Published:** 2017-02-25

**Authors:** Xiaoyong Li, Junping Ao, Ji Wu

**Affiliations:** ^1^ Renji Hospital, Key Laboratory for the Genetics of Developmental and Neuropsychiatric Disorders (Ministry of Education), Bio-X Institutes, School of Medicine, Shanghai Jiao Tong University, Shanghai, 200240, China; ^2^ State Key Laboratory of Oncogenes and Related Genes, Shanghai Cancer Institute, Renji Hospital, Shanghai Jiao Tong University School of Medicine, Shanghai, 200032, China; ^3^ Key Laboratory of Fertility Preservation and Maintenance of Ministry of Education, Ningxia Medical University, Yinchuan, 750004, China; ^4^ Shanghai Key Laboratory of Reproduction Medicine, Shanghai, 200025, China

**Keywords:** spermatogonial stem cell, female germline stem cell, lncRNA, circRNA, ceRNA

## Abstract

Accumulating evidence indicates that long noncoding RNAs (lncRNAs) and circular RNAs (circRNAs) involve in germ cell development. However, little is known about the functions and mechanisms of lncRNAs and circRNAs in self-renewal and differentiation of germline stem cells. Therefore, we explored the expression profiles of mRNAs, lncRNAs, and circRNAs in male and female mouse germline stem cells by high-throughput sequencing. We identified 18573 novel lncRNAs and 18822 circRNAs in the germline stem cells and further confirmed the existence of these lncRNAs and circRNAs by RT-PCR. The results showed that male and female germline stem cells had similar GDNF signaling mechanism. Subsequently, 8115 mRNAs, 3996 lncRNAs, and 921 circRNAs exhibited sex-biased expression that may be associated with germline stem cell acquisition of the sex-specific properties required for differentiation into gametes. Gene Ontology (GO) and KEGG pathway enrichment analyses revealed different functions for these sex-biased lncRNAs and circRNAs. We further constructed correlated expression networks including coding–noncoding co-expression and competing endogenous RNAs with bioinformatics. Co-expression analysis showed hundreds of lncRNAs were correlated with sex differences in mouse germline stem cells, including lncRNA Gm11851, lncRNA Gm12840, lncRNA 4930405O22Rik, and lncRNA Atp10d. CeRNA network inferred that lncRNA Meg3 and cirRNA Igf1r could bind competitively with miRNA-15a-5p increasing target gene Inha, Acsl3, Kif21b, and Igfbp2 expressions. These findings provide novel perspectives on lncRNAs and circRNAs and lay a foundation for future research into the regulating mechanisms of lncRNAs and circRNAs in germline stem cells.

## INTRODUCTION

Germline stem cells belong to adult stem cells, and they possess the ability that transmit genetic information from generation to generation [1–5]. Mammalian male germline stem cells, also know as spermatogonial stem cells (SSCs), were identified in the 1950s [2, 6, 7]. With the development of the transplantation technique [8, 9], *in vitro* SSCs culture systems [10, 11], and traditional methods, the study of SSCs has advanced to include molecular mechanisms and signal transduction.

A new class of germ cells, female germline stem cells (FGSCs), has been successfully isolated and purified using mouse vasa homolog (MVH)-based immunomagnetic sorting from neonatal and adult mammalian ovaries [6, 7, 12]. Although, compared with SSCs, less is known about FGSCs, an increasing number of research is now being focused on FGSCs [13, 6, 14]; in particular, the isolation and characterization of FGSCs from rat and human ovaries have allowed their biological functions and applications to be studied further [7, 15–17].

There is growing recognition that cells, especially mammalian cells, produce thousands of large noncoding transcripts. Long noncoding RNAs (lncRNAs) are a class of nucleic acid molecules defined as transcripts longer than 200 nucleotides (nt) that lack significant ORF (open reading frames) [18, 19]. LncRNAs are involved in a variety of biological processes, including maintenance of genome integrity, stem cell pluripotency, genomic imprinting, X inactivation, cell differentiation [19–26]. Circular RNAs (circRNAs) are a newly identified type of noncoding RNAs that is characterized by the presence of a covalent bond linking the 3′ and 5′ ends generated by backsplicing [19, 27–33]. CircRNAs are expressed widely in tissue- and developmental stage-specific patterns and a subset of circRNAs are conserved across species [32–41]. Currently, the functional research of circRNAs are mainly focused on microRNA sponges, RNA-binding protein and nuclear transcriptional regulators [33, 37, 42–45]. However, we know very little about the functions and mechanisms of lncRNAs and circRNAs in germline stem cells. Therefore, it is significant to study the transcription and functions of lncRNAs and circRNAs in germline stem cells, because the results may contribute to an understanding of their roles in reproduction and development.

Currently, we investigated the expression profiles of lncRNAs and circRNAs in male and female mouse germline stem cells by high-throughput sequencing. We identified 18573 novel lncRNAs and 18822 circRNAs and further confirmed the existence of these lncRNAs and circRNAs using qRT-PCR and RT-PCR. The whole gene expression profiles of SSCs and FGSCs showed that certain genes had similar gene expression patterns at both the mRNA and lncRNA levels. Further, we showed that FGSCs had similar GDNF signaling mechanism as SSCs. We also investigated the sex-biased lncRNAs, mRNAs, and circRNAs in SSCs and FGSCs using high-throughput sequencing. We not only detected associated gene ontology (GO) terms and kyoto encyclopedia of genes and genomes (KEGG) pathways, but also delineated comprehensive functional landscapes of coding–noncoding co-expression and competing endogenous RNAs (ceRNAs) in germline stem cells using bioinformatics approaches. Our findings reveal, for the first time, lncRNA and circRNA profiles related to the self-renewal and the sex-specific properties required for differentiation into gametes and provide insights into sex differences in lncRNA and circRNA expression in germline stem cells, which could promote studies of their roles in germline stem cells.

## RESULTS

### Strand-specific RNA sequencing and assembly of mouse germline stem cell libraries

**Figure 1:**
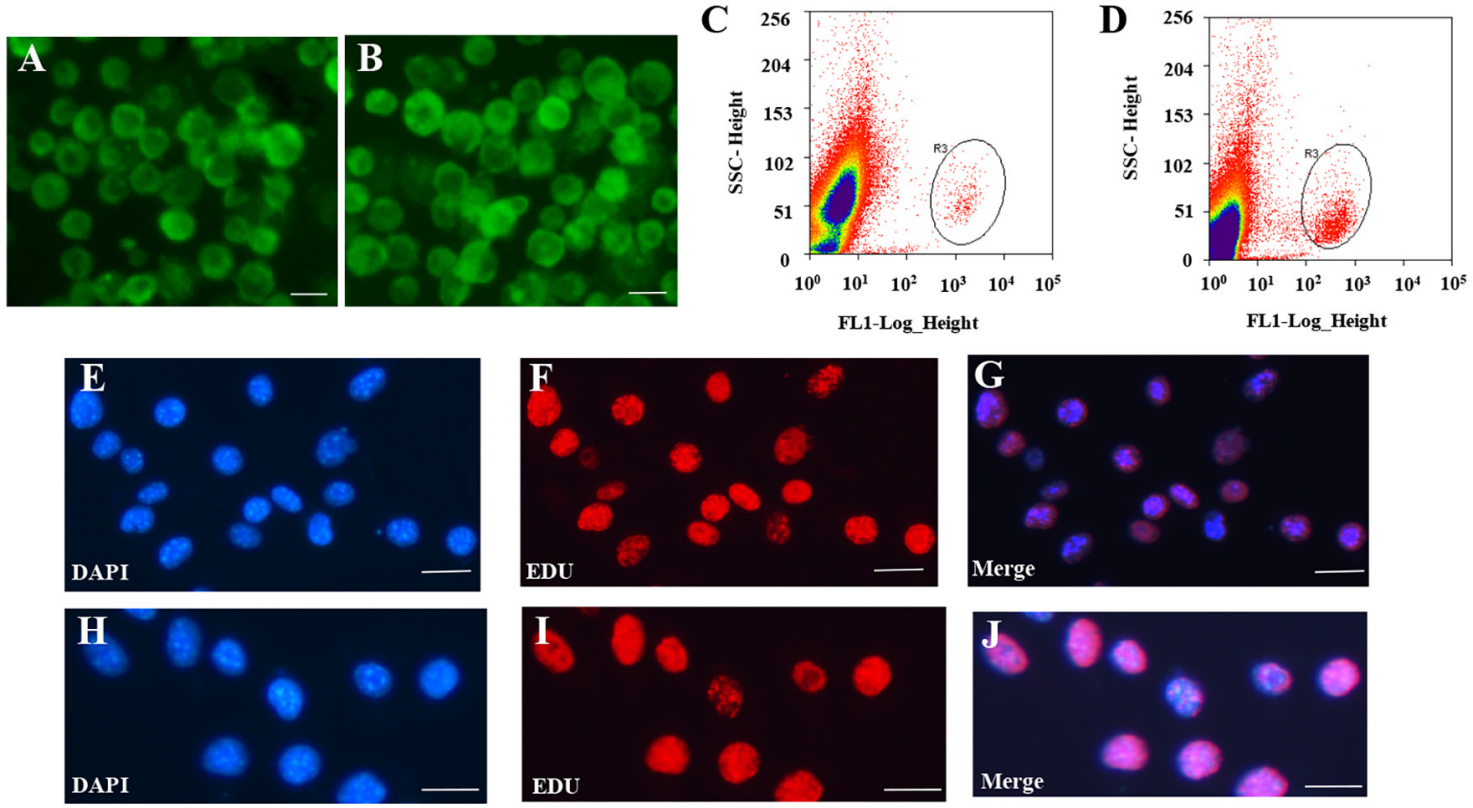
Isolation and purification of FGSCs and SSCs (**A**–**B)** Representative morphology of female germ line stem cells (FGSCs) (A) and male germ line stem cells (SSCs) (B) under fluorescence microscopy after purification with fluorescence-activated cell sorting (FACS). (**C**–**D)** Representative examples of FGSC (C) and SSC purification (D) with FACS. (**E**–**G**) Purity of FGSCs was evaluated by immunofluorescence analysis of 5-ethynyl-2′-deoxyuridine (EdU). (**H**–**J**) Purity of SSCs was evaluated by immunofluorescence analysis of EdU. Scale bars = 10 μm. Each experiment was conducted three times.

For systematic identification and comparison of the expression patterns of lncRNAs and circRNAs with associated co-expression and ceRNA networks in mouse germline stem cells (Supplementary Figure 1), we isolated SSCs and FGSCs using two-step enzymatic digestion, as described previously [10, 12]. The cells were purified using fluorescence-activated cell sorting (see MATERIAL AND METHODS, Figure 1A–1D). The germline stem cells were evaluated by the following experiments. Firstly, we determined the expression of Mvh (mouse vasa homolog, also termed DEAD box polypeptide 4, Ddx4) [46], Dazl (deleted in azoospermia-like) [47, 48], Fragilis (also termed interferon induced transmembrane protein 3, Ifitm3) [49], Oct4 (organic cation/carnitine transporter4, also termed POU domain, class 5, transcription factor 1, Pou5f1) [50], Stella (also termed developmental pluripotency-associated 3, Dppa3) [49, 51] and Blimp1 (also termed PR domain containing 1, with ZNF domain, Prdm1) [52] in SSCs and FGSCs that we isolated using RT-PCR analysis. The results showed that the cells express Mvh, Dazl, Fragilis, Oct4, Stella and Blimp1 (Supplementary Figure 2A). Secondly, immunofluorescence analysis confirmed the expression of Mvh, Oct4 and Dazl (Supplementary Figure 2B and 2C). Thirdly, based on the results from our previous study, we further checked eighteen stemness genes expression in isolated SSCs and FGSCs [6]. Using RT-PCR analysis, all of the eighteen stemness genes were expressed in both isolated SSCs and FGSCs (Supplementary Figure 2D). And lastly, immunofluorescence analysis of EdU (5-ethynyl-2′-deoxyuridine) incorporation demonstrated that both isolated SSCs and FGSCs possessed proliferative ability and SSCs and FGSCs that we isolated contained same portion (> 90%) of the truly germlien stem cells (Figure 1E–1J). All the characteristics detected clearly demonstrate both SSCs and FGSCs that we isolated possessed germ cell and stem cell characteristics. Using Illumina (paired-end) sequencing technology to analyze the expression profiles of mRNAs and non-coding RNAs in the two types of germline stem cells. A total of 249,912,216 and 261,924,516 raw reads were generated in the SSC and FGSC libraries, respectively. The GC content was 53.5% and 50.7% in the two libraries, respectively. After discarding reads with adapters, poly-N >10%, and other possible contaminants, 233,978,622 (93.6%) and 246,157,442 (94.0%) clean reads remained and were used in the following analyses. Approximately 86.7% and 91.1% of the clean reads in the SSC and FGSC libraries, respectively, were mapped to the mouse reference genome (UCSC mm10) [53], and 58364 transcripts were assembled using Scripture [54] and Cufflinks [55] (Table 1).

**Table 1:** Alignment and quantification statistics in each strand-specific RNA-seq library sample

Library	Total reads	Number of reads after trim	Percentage trimmed	Percentage aligned	Percentage uniquely aligned
FGSC lib-1	87193802	81770884	93.78	90.81	80.69
FGSC lib-2	87466014	82531960	94.36	90.14	81.12
FGSC lib-3	87264700	81854598	93.80	92.35	83.98
SSC lib-1	76414038	71636014	93.75	86.81	77.18
SSC lib-2	87385442	81823768	93.64	87.56	78.40
SSC lib-3	86112736	80518840	93.50	85.72	75.47

### Novel lncRNAs were identified in both SSCs and FGSCs

**Figure 2:**
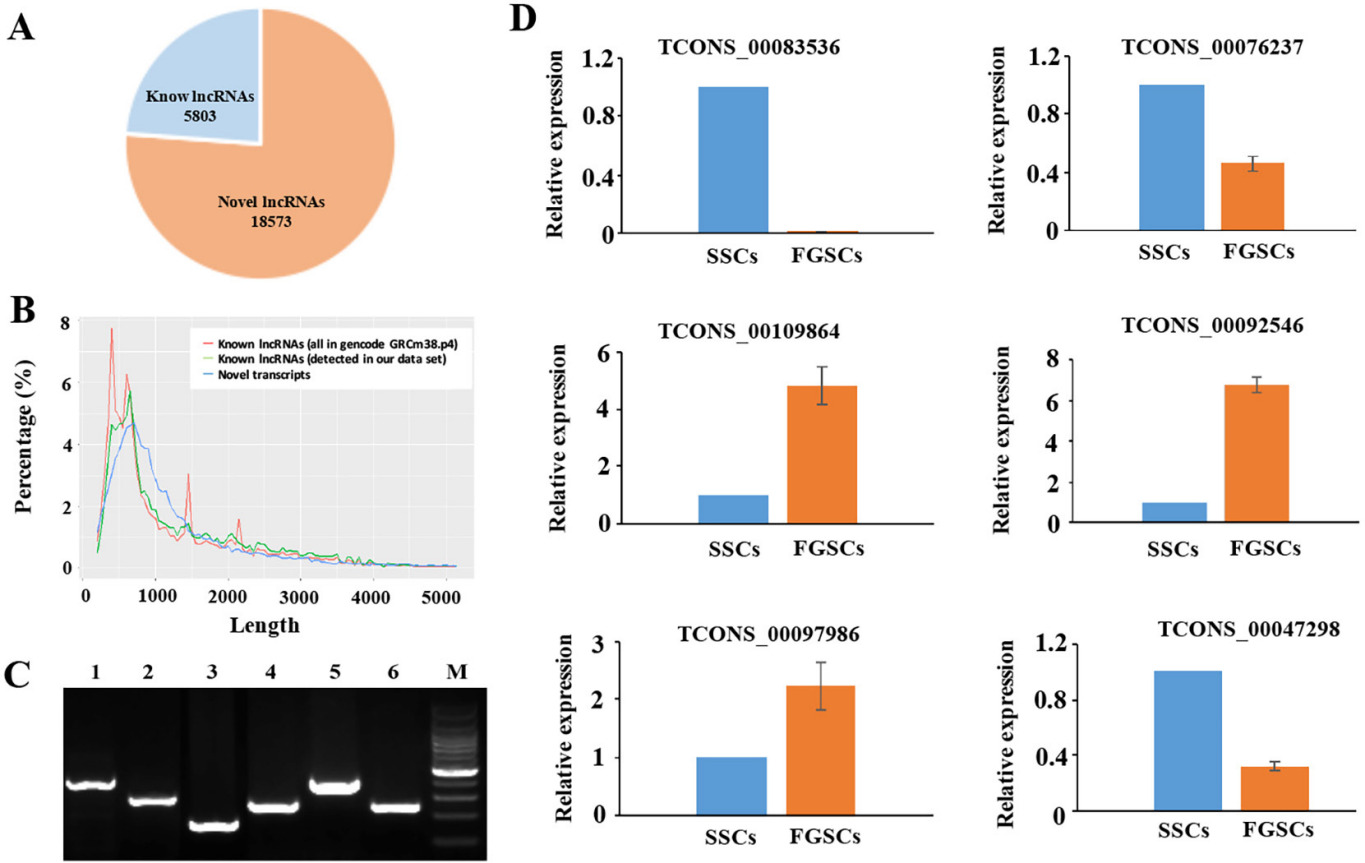
Novel LncRNAs were identified and verified (**A**) A pie diagram showing the number of novel and known lncRNAs we identified. (**B**) Length distribution of the potential novel lncRNA transcripts. The known lncRNAs in NONCODE v3.0 database are used as a control (both the whole data set and the ones detected in our RNA- Seq data). (**C**) Validation of 6 randomly selected novel lncRNAs by RT-PCR. Lanes 1–6 randomly selected novel lncRNAs (TCONC_00109864, TCONC_00083536, TCONC_0047298, TCONC_00076237, TCONC_00097986, TCONC_00092546); M, 100bp DNA ladder. (**D**) Expression patterns of the selected lncRNAs in the two groups were consistent with FPKM values of these lncRNAs, and the sequencing results correlated with the qRT-PCR results. GAPDH was used as internal control. The experiments were conducted three times.

We searched across all six samples (see MATERIAL AND METHODS) for novel lncRNAs that were not present in the RefSeq (https://www.ncbi.nlm.nih.gov/refseq/), Ensembl (http://asia.ensembl.org/index.html), or Noncode v3.0 (http://www.noncode.org/index.php) lncRNA databases. To minimize the false-positive rates in identifying novel lncRNAs from among the 58364 assembled transcripts, we developed a stringent filtering pipeline to discard transcripts without all the characteristics of lncRNAs. After discarding transcripts that were less than 200 bp in length and had one exon, there-read coverage, we evaluated the coding potential of the remaining transcripts using the CPC (Coding Potential Calculator) and CNCI (Coding-Non-Coding Index) software [56], and identified 18573 possible novel lncRNAs (Supplementary Table 1) and 5803 known lncRNAs (Supplementary Table 2) (Figure 2A).

The average length of the novel lncRNAs was 1427 nucleotides, similar to the known lncRNAs (Figure 2B). The average size of the open reading frames (ORFs) in the lncRNAs and mRNAs was 86.24 bp and 394.84 bp, respectively, which indicated that the mRNA ORFs were significantly longer than lncRNAs ORFs. LncRNAs cannot code for proteins because they lack significant ORFs. Among the 18573 novel lncRNAs, we found 4300 that were expressed at an FPKM (fragments per kilobase of exon per million reads mapped) value of > 1 and 12022 that were expressed at an FPKM value of > 0.5. A total of 18336 novel lncRNAs were expressed at an FPKM value of > 0.1 in at least one of the two samples.

Six putative novel lncRNA transcripts identified in SSCs and FGSCs libraries were randomly selected to validate using RT-PCR. As shown in Figure 2C, six putative lncRNA transcripts were all amplified with expected size. In addition, we selected TCONS_00083536, amplified by RT-PCR, to clone its sequence using RACE clone technology, and a 1163 bp length sequence was cloned. The cloned sequence can blast with the RNA-seq data completely. The qRT-PCR results indicated that the expression patterns of the selected lncRNAs in the two groups were consistent with FPKM values of these lncRNAs, and the sequencing results correlated with the qRT-PCR results (Figure 2D). All of these above mentioned showed that our pipeline is highly strict in identifying putative lncRNA, and most of them are truly expressed *in vivo*.

### Chromosomal location and classification of the novel lncRNAs

**Figure 3:**
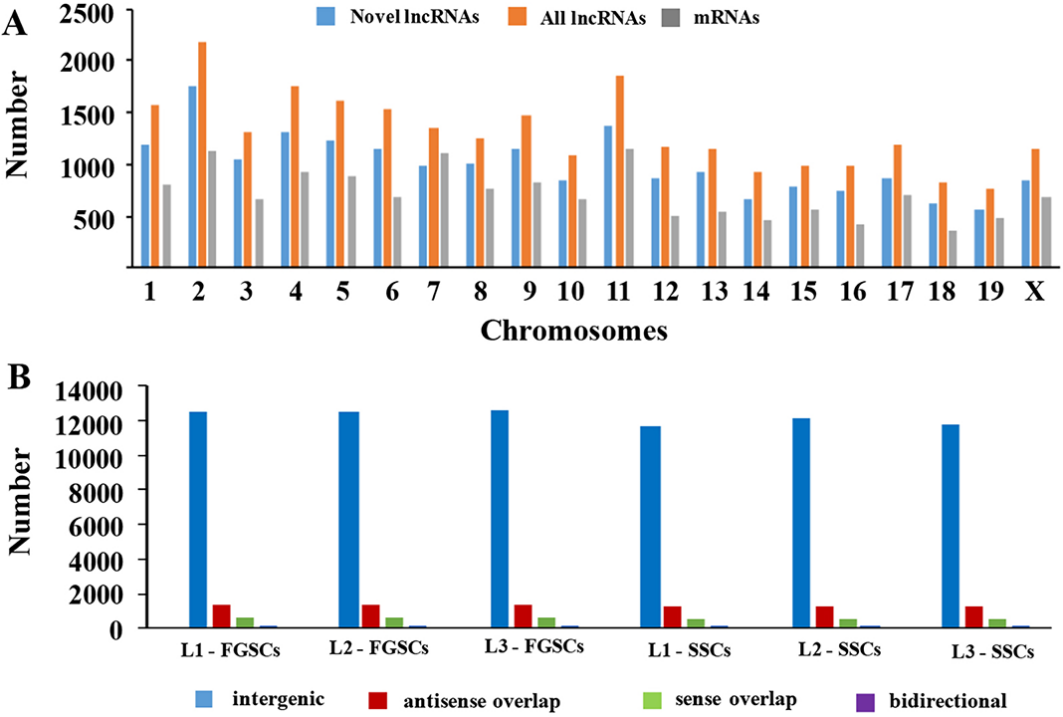
Chromosomal distribution and classification of the novel lncRNAs (**A**) The numbers of lncRNAs and mRNAs localized on each chromosome. (**B**) The numbers of the four types of novel lncRNAs (sense, antisense, bidirectional, and intergenic) that existed in the male and female germline stem cells (SSCs and FGSCs).

The lncRNA and mRNA transcripts were found to be distributed on all of the mouse chromosomes. Statistical analysis showed that the novel lncRNAs were widely scattered in all the chromosomes and that the ratio of lncRNA expression was much higher than mRNA expression from each chromosome (Figure 3A). These results demonstrated that transcription of lncRNA genes were in accordance well with the transcription of mRNA genes in both the male and female germline stem cells.

In addition, according to its location relative to nearby protein-coding genes, a lncRNA could be classified as sense overlap lncRNA, bidirectional lncRNA, antisense lncRNA, or intergenic lncRNA [57–61]. Among the novel lncRNAs in our study, the intergenic lncRNAs constituted the majority, and the numbers of lncRNAs of each type were similar between SSCs and FGSCs (Figure 3B).

### Similar lncRNA profiles and GDNF signaling mechanism shared by mouse SSCs and FGSCs

**Figure 4:**
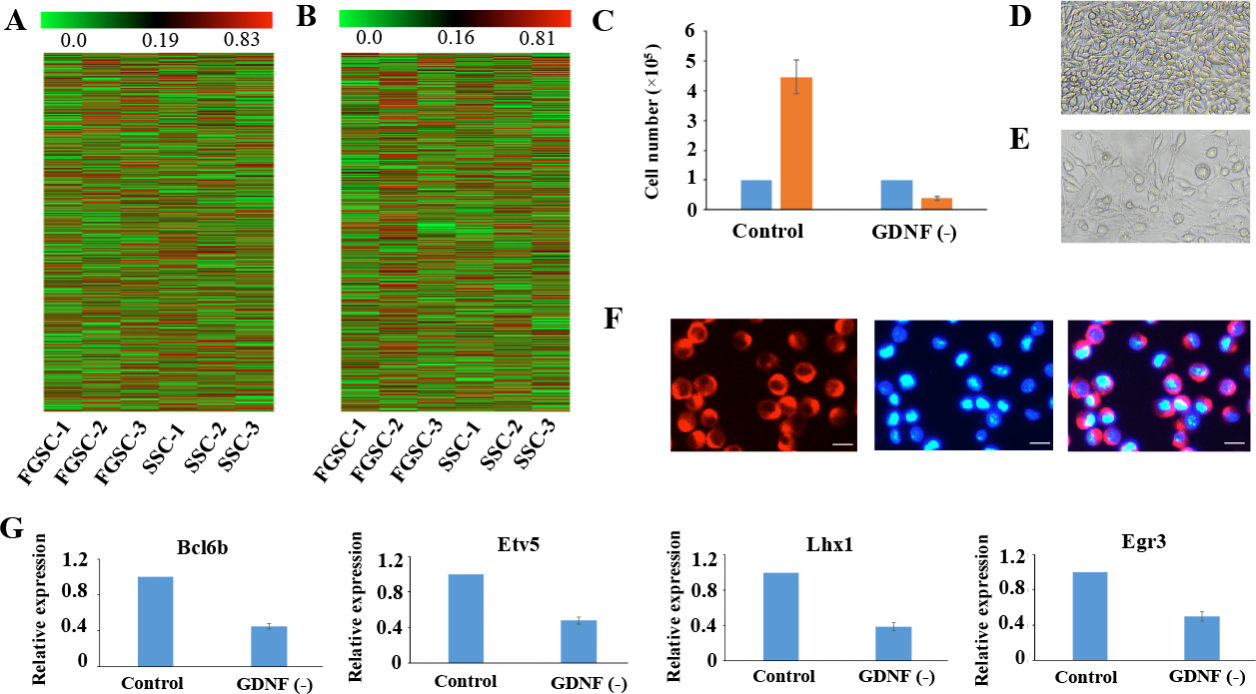
Similar lncRNA profiles and self-renewal mechanisms shared by mouse SSCs and FGSCs (**A**–**B**). Heat map showing expression profiles of mRNAs (A) and lncRNAs (B). The maps are based on the expression values of all expressed lncRNAs and mRNAs detected by high-throughput sequencing. The color scale indicates the expression values; the intensity increases from green to red. Each column represents one sample, and each row represents a transcript. (**C**–**E**) Proliferation of FGSCs in culture was dependent on the glial cell line-derived neurotrophic factor GDNF. Cells plated at 1.0 × 10^5^ per well into culture medium without GDNF did not proliferate (average 0.4 × 10^5^ per well) after a week. FGSCs were cultured with culture medium containing GDNF as the control. Error bars indicate the standard deviation (SD). Each experiment was conducted three times. (**F**) The FGSCs was detected by immunofluorescence analysis with the antibodies against GFRA1. Left, GFRA1 immunofluorescence. Middle, DAPI immunofluorescence. Right, merge for GFRA1 and DAPI immunofluorescence Scale bars: 10 μm. (**G**) Withdrawal of GDNF for a week resulted in a significant change in the expression levels of self-renewal-related genes that showed the largest response to the GDNF signal, including Bcl6b, Etv5, Lhx1, and Egr3. GAPDH was used as an internal control. Error bars indicate the standard deviation (SD). Each experiment was conducted three times.

Our previous microarray data showed that the whole gene expression profiles of SSCs and FGSCs were similar [6]. Here, our high-throughput sequencing data gave the same results; that is, the whole gene expression patterns showed that certain genes had similar expressing patterns at the mRNA and lncRNA levels in both SSCs and FGSCs (Figure 4A, 4B). In addition, functional analysis of the highly co-expressed mRNAs and lncRNAs showed that they were enriched in gene ontology (GO) terms related to cell cycle, cell proliferation and cell division (Supplementary Figure 3), implying that SSCs and FGSCs had similar germline stem cell maintenance mechanisms, consistent with the results of our previous research with microarray data [6].

GDNF is the first priority extrinsic factor that promotes self-renewal of SSC in a dose-dependent patter. GDNF signals promote self-renewal of SSC by PI3K-Akt, Ras/ERK1/2 and SFK pathways. [7, 62, 63]. Interestingly, the strong enrichment PI3K-Akt pathway, which was observed in the highly co-expressed mRNAs and lncRNAs of SSCs and FGSCs (Supplementary Figure 4), suggests that FGSCs had a similar GDNF signaling mechanism as SSCs. To determine whether FGSCs had the same GDNF signaling mechanism as SSCs, we removed GDNF from the culture medium for a period of 7 days and found that the results were similar to those of a previous study on SSCs [64]. Withdrawal of GDNF for a week resulted in a significant reduction in cell numbers (of 1.0 × 10^5^ plated cells, an average of 0.4×10^5^ remained after GDNF depletion) (Figure 4C–4E). Subsequently, we showed that GFRα1 (GDNF family receptor alpha 1) expressed on the surface of FGSCs by immunofluorescence staining (Figure 4F). A previous study showed that the genes that responded most dramatically to GDNF may be downstream effectors of GDNF signals, such as Bcl6b, Lhx1, Etv5, and Egr3 [7, 65]. In view of the above information, we further found withdrawal of GDNF for a week resulted in decrease in the gene expression levels of these self-renewal-related genes that responded most to GDNF signaling in FGSCs (Figure 4G). These data indicate that FGSCs has similar GDNF signaling mechanism as SSCs.

### Transcriptome analysis reveals abundant lncRNAs with sex-biased expression

Germline stem cells are derived from primordial germ cells and are subsequently subjected to a sex-specific fate to become male and female germline stem cells [3]. We have shown that similar lncRNA profiles and self-renewal mechanisms are shared by mouse SSCs and FGSCs. However, the sex-specific basis of germline stem cells is poorly understood.

**Figure 5:**
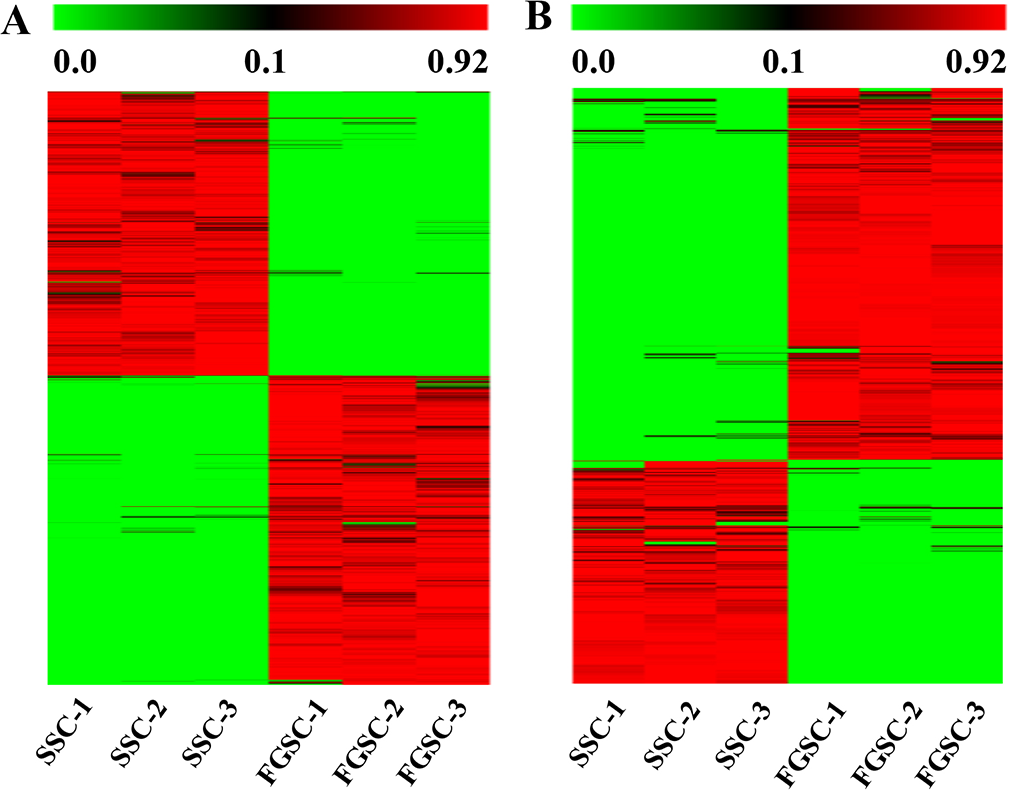
Transcriptome profiling reveals abundant mRNAs (**A**) and lncRNAs (**B**) with sex-biased expression. The maps correspond to normalized expression values of significantly changed lncRNAs and mRNAs with fold change ≥ 2.0, *p-value* < 0.05, and FDR < 0.05. The color scale indicates the expression values; the intensity increases from green to red. Each column represents one sample, and each row represents a transcript.

Accurate gene expression profiles have been provided by our strand-specific RNA sequencing, consequently, we were able to select genes with sex-biased expression in male and female germline stem cells for analysis of sex specifically expression. According to the lncRNA and mRNA expression profiles, 8115 mRNAs and 3996 lncRNAs (including 3695 novel lncRNAs) exhibited sex-biased expression (absolute *p*-value < 0.05) between male and female germline stem cells. Of these sex-biased lncRNAs and mRNAs, 1500 lncRNAs (including 1364 novel lncRNAs) and 4221 mRNAs exhibited male-biased expression and 2496 lncRNAs (including 2331 novel lncRNAs) and 3894 mRNAs exhibited female-biased expression (Figure 5A, 5B). The Fisher's exact test was applied to evaluate the chromosomal distribution for the sex-biased lncRNAs and mRNAs by calculating odds ratio [66]. The odds ratio was defined as the ratio between male-biased ncRNAs and mRNAs (autosomal/X-linked) and non-male-biased lncRNAs and mRNAs (autosomal/X-linked) respectively [66]. Therefore, an odds ratio >1 indicates male-biased genes are enriched on autosomes and an odds ratio <1 indicates X enrichment. The odds ratio for male-biased lncRNAs was 1.22 (*p* < 0.01, Fisher's exact test for each comparison), indicating that male-biased lncRNAs were enriched on autosomes. A similar trend was found for mRNAs, the odds ratio for male-biased mRNAs was 1.41. In contrast, we found that female-biased lncRNAs and mRNAs were significantly overrepresented on the X chromosomes in SSC versus FGSC comparisons (the odds ratios were 0.81 and 0.74, respectively). The results showed that thousands of lncRNAs and mRNAs exhibited sex-biased expression profiles and these sex-biased lncRNAs and mRNAs were non-randomly distributed between the X chromosome and autosomes.

### Target prediction for sex-biased lncRNAs and function analysis

**Figure 6:**
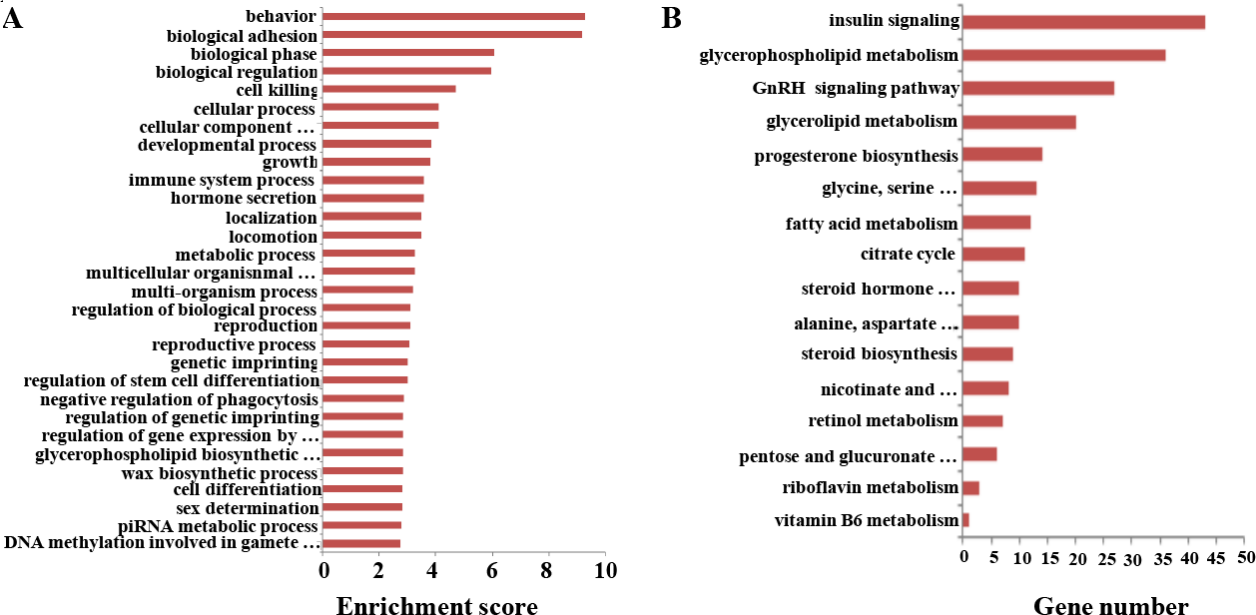
Gene Ontology (GO) and KEGG pathway analyses of sex-biased lncRNAs (**A**) GO annotations of the sex-biased lncRNAs showing the 30 enrichment scores for terms under the biological process category. A full list of the assigned GO terms is given in Supplementary Table 3. (**B**) Enriched KEGG pathway terms related with the sex-biased differentiation potential between the male and female germline stem cells. A full list of the KEGG terms is given in Supplementary Table 4.

As mentioned previously, lncRNAs are usually coordinately transcribed with their associated mRNAs and could regulate the transcription of their overlapping or adjacent mRNAs in numerous ways [19]. To a certain extent, the functions of lncRNAs could be mirrored through their associated mRNAs by cis-regulation and trans-regulation [19]. Therefore, the functions of the sex-biased expressed lncRNAs were predicted based on the GO and KEGG pathway annotations of their target genes [67]. In the GO analysis over-represented terms were identified under the three main GO categories, including biological process, molecular function, and cellular component. In the GO analyses, the most frequently predicted functions of lncRNAs were associated mainly with behavior, biological adhesion, biological phase, and biological regulation. The functional clusters were shown in Figure 6A, a full list of the assigned GO terms is shown in Supplementary Table 3. Interestingly, the sex-specific mRNAs and lncRNAs were enriched in GO terms related to genetic imprinting and regulation of genetic imprinting, which indicated that differences in genetic imprinting may be possible regulate the expression of sex-specific genes in SSCs and FGSCs.

To obtain further perceptions of the different biological functions of mRNAs and lncRNAs in SSCs and FGSCs, we performed KEGG pathway analysis [3]. A total of 3996 sex-biased expressed lncRNAs in the SSC versus FGSC comparison were assigned to 204 KEGG pathways (Figure 6B, Supplementary Table 4). Interestingly, some of identified KEGG pathways were involved in glycometabolism, protein metabolism, and lipid metabolic pathways, and included steroid biosynthesis, glycerophospholipid metabolism, fatty acid metabolism, citrate cycle, glycerolipid metabolism, retinol metabolism, pentose and glucuronate interconversions. The main difference between SSCs and FGSCs was the sex-specific properties required for differentiation into germ cells; SSCs differentiate into sperm while FGSCs differentiate into oocytes. Our results suggest that glycometabolism, protein metabolism, and lipid metabolic pathways occupy a significant position in cell differentiation processes, and lncRNAs may influence their sex-specific properties through these signaling pathways.

Strong enrichment was observed in hormone-related signaling pathways, such as progesterone biosynthesis, steroid hormone biosynthesis, GnRH signaling, and insulin signaling pathways, as well as vitamin-related signaling pathways, such as vitamin B6 metabolism. Because hormones and vitamins often act as inducers in the SSC and FGSC differentiation processes, these signaling pathways may be the main regulating pathways in sex-specific gene expression, leading to the sex-specific properties of SSCs and FGSCs. In particular, sex hormones are quite different between male and female cells. The enriched GO terms and KEGG pathways revealed here offer new viewpoints on the typical characteristics of SSCs and FGSCs.

### Co-expression of sex-biased lncRNAs and mRNAs and function prediction

**Figure 7:**
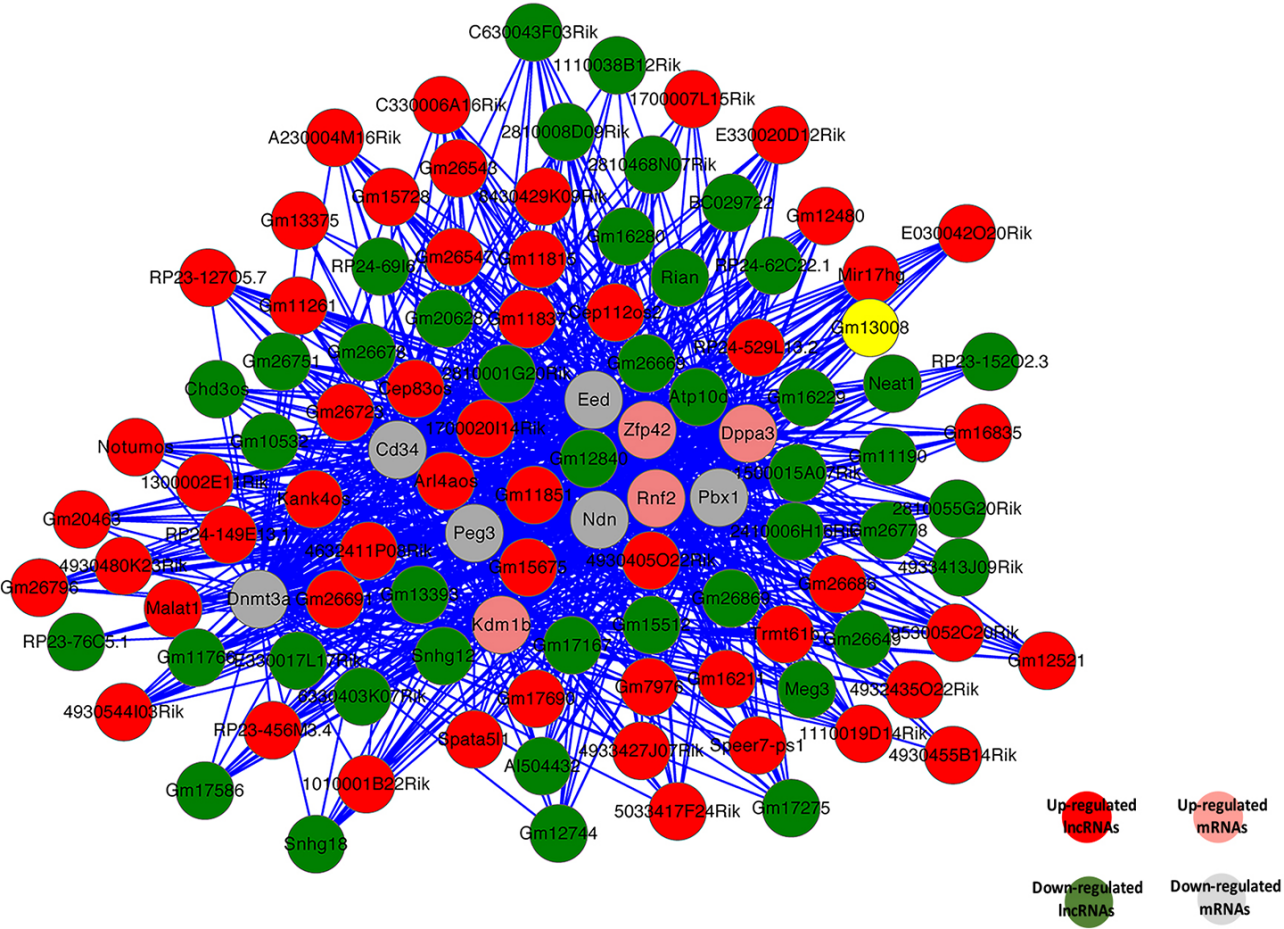
Co-expression network of 10 significant mRNAs with their associated lncRNAs The co-expression network is based on the Pearson correlation coefficient (the absolute value of PCC ≥ 0.99, *p*-value < 0.01, and FDR < 0.01). Solid lines indicate positive correlations; dashed lines indicate negative correlations.

Up to now, most lncRNAs have not been functionally annotated; therefore, the prediction of lncRNA functions has been based on the annotations of the co-expressed mRNAs [68]. We constructed the coding-non-coding gene co-expression network (CNC network) based on the correlation analysis between sex-biased expressed lncRNAs and mRNAs [69, 70]. We choose top 10 significantly sex-biased expressed coding genes in SSCs and FGSCs to build the CNC network (Figure 7). These mRNAs involved in numbers of biological processes, including reproduction, regulation of stem cell differentiation, reproductive process, regulation of genetic imprinting, genetic imprinting, cell differentiation, sex differentiation, regulation of gene expression by genetic imprinting, and sex chromatin. The constructed network showed that upregulated lncRNA Gm11851 was negatively correlated and downregulated lncRNA Gm12840 was positively correlated with Eed, Ndn, and Peg3, which are involved in genetic imprinting; whereas, upregulated lncRNA 4930405O22Rik was positively correlated and downregulated lncRNA Atp10d was negatively correlated with Zfp42, Dppa3, and Rnf2, which are associated with regulation of genetic imprinting. As previous studies, our CNC network also suggested that one mRNA was correlated with one to ten lncRNAs [69]. More importantly, the co-expression network indicateds the mechanism of lncRNAs and mRNAs in germline stem cell acquisition of the sex-specific properties required for differentiation into gametes

### Identification and functional analysis of circRNAs in germline stem cells

**Figure 8:**
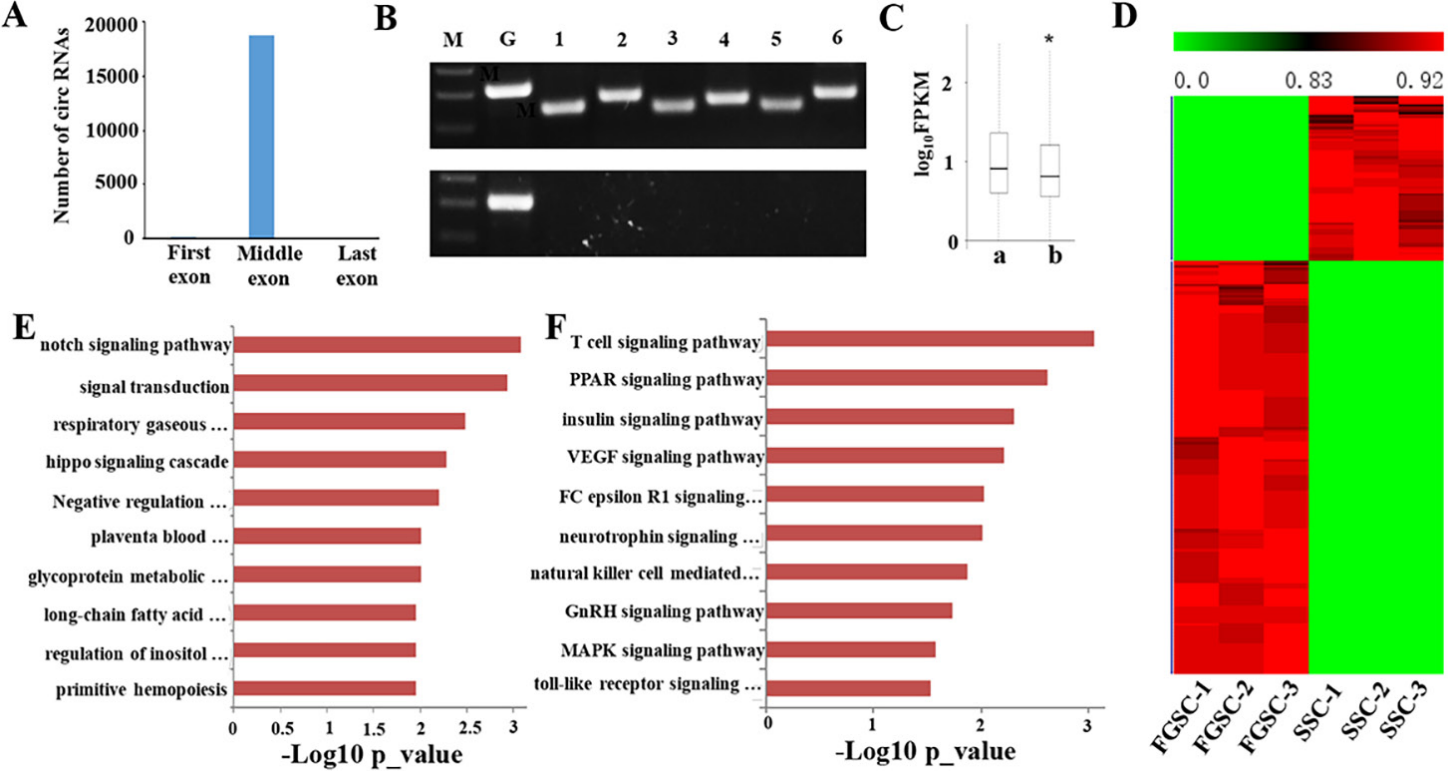
Identification and functional analysis of circRNAs in germline stem cells (**A**) Distribution of the backspliced exons in circRNAs. Nearly all (99.9%) backspliced exons that contribute to circRNAs are located in the middle of their hosting genes; 19 are in the first exon and none are in the last exon. (**B**) Six candidate circRNAs were tested for the presence/absence of a poly-A tail using either oligo (dT) (bottom band) or random hexamers (upper band) to amplify the total RNA followed by a RT-PCR assay with primers specific for backsplice junctions of candidate circRNAs and for the linear mRNAs of β-actin and GAPDH. While both the oligo (dT) and random hexamers amplified the GAPDH mRNA, only the random hexamers produced PCR products for the six tested candidate circRNAs. M, Maker; G, GAPDH; 1–6, candidate circRNAs. (**C**) Comparison of the expression levels of circRNA hosting genes and other coding genes in male and female germline stem cells. The expression levels of the hosting genes only include linear transcripts by excluding circular transcripts. a., the expression levels of circRNA hosting genes. b., the expression levels of other coding genes. (*p-value < 0.05, student's *t*-test). (**D**) Transcriptome profiling revealed abundant circRNAs with sex-biased expression. The maps correspond to normalized expression values of significantly changed circRNAs with fold change ≥ 2.0, *p*-value < 0.05, and FDR < 0.05. The color scale indicates the expression values; the intensity increases from green to red. Each column represents one sample and each row represents a transcript. (**E**) GO analyses of sex-biased circRNAs showing the top 10 enrichment scores for terms under the biological process category. A full list of the assigned GO terms is given in Supplementary Table 6. (**F**) KEGG pathway analyses of sex-biased lncRNAs with the top 10 enrichment scores. A full list of the KEGG terms is given in Supplementary Table 7.

Recently, tens of thousands of circRNA genes with various functional roles have been identified in the mouse genome [33, 71–74]. We used the CIRI software to analyze the RNA seq reads for the existence of circRNAs in germline stem cells [75]. To exclude false-positive candidates, we manually filtered out circRNA candidates with junction regions spanning over two genomic contigs as well as publicly available linear nucleotide sequences [75]. A total of 18822 circRNAs derived from 5334 hosting genes were identified in the mouse germline stem cells (Supplementary Table 5). Most of the 18822 identified circRNAs were exonic circRNAs, and only 345 were intronic circRNAs. We found that 9812 (52.13%) circRNAs are derived from the sense strand and 9010 (47.87%) circRNAs were derived from the antisense strand. A previous study showed that circRNAs usually lacked of the first and last exons of their hosting genes [76], and we acquired similar results in mouse germline stem cells: 18803 (99.9%) of the 18822 circRNAs had missed the first or last exons of their hosting genes [77] (Figure 8A).

To confirm the candidate circRNAs are not misidentified linear products of trans-splicing events, we designed multiple pairs of outward facing primers for representative circRNAs to amplify the backsplice exon junction from cDNA of the SSCs and FGSCs when the cDNA was created by priming with random hexamer primers. Each primer pair produced a single distinct band with the expected product size in an RT-PCR assay, indicating the presence of the circular junction in the SSC and FGSC samples (Figure 8B). Furthermore, when cDNA was created by priming with oligo (dT) primers, it is expected that only poly-adenylated RNAs will be amplified. This cDNA did indeed fail to produce any amplification products for the circRNA candidates in the RT-PCR assay (Figure 8B). These results strongly indicate the absence of a poly-A tail for the candidate circRNAs.

Next, as previous study, we compared the expression levels of circRNA hosting genes with others, the results showed that the averaged expression levels of circRNA hosting genes were significantly higher than the genes that no detectable circular transcripts in both SSCs and FGSCs [77] (Figure 8C). To predict the function of these circRNA hosting genes in germline stem cells, we performed GO and pathway analysis. The results showed that circRNA hosting genes were expressed in cell specific pattern. Most of GO terms and KEGG pathways for circRNA hosting genes in germline stem cells were mainly involved in self- renewal and differentiation of germline stem cells, such as reproduction, genetic imprinting, reproductive process, stem cell maintenance, cell differentiation, germ cell development, stem cell division, TGF-beta signaling pathway and PI3K-Akt signaling pathway (Supplementary Figures 5 and 6).

The circRNA expression profiles showed that 921 circRNAs exhibited sex-biased expression (absolute *p*-value < 0.05) between the male and female germline stem cells. Of these sex-biased circRNAs, 245 displayed male-biased expression and 676 displayed female-biased expression (Figure 8D). Furthermore, we separated all 921 sex-biased circRNA host genes in SSCs and FGSCs. The GO and KEGG pathway analyses revealed different functions for the identified sex-biased circRNAs that were associated with acquisition of the sex-specific properties required for differentiation into germ cells [3] (Figure 8E and 8F). The top 10 GO terms are shown in Figure 8E and a full list of the GO terms is given in Supplementary Table 6. The GO terms include notch signaling pathway, negative regulation of cell differentiation, signal transduction and long-chain fatty acid biosynthesis. The top 10 KEGG pathways are shown in Figure 8F show and a full list of the KEGG pathways is given in Supplementary Table 7. The KEGG pathways include PPAR signaling pathway, insulin signaling pathway, VEGF signaling pathway, and GnRH signaling pathway.

### Construction of ceRNA network

**Figure 9:**
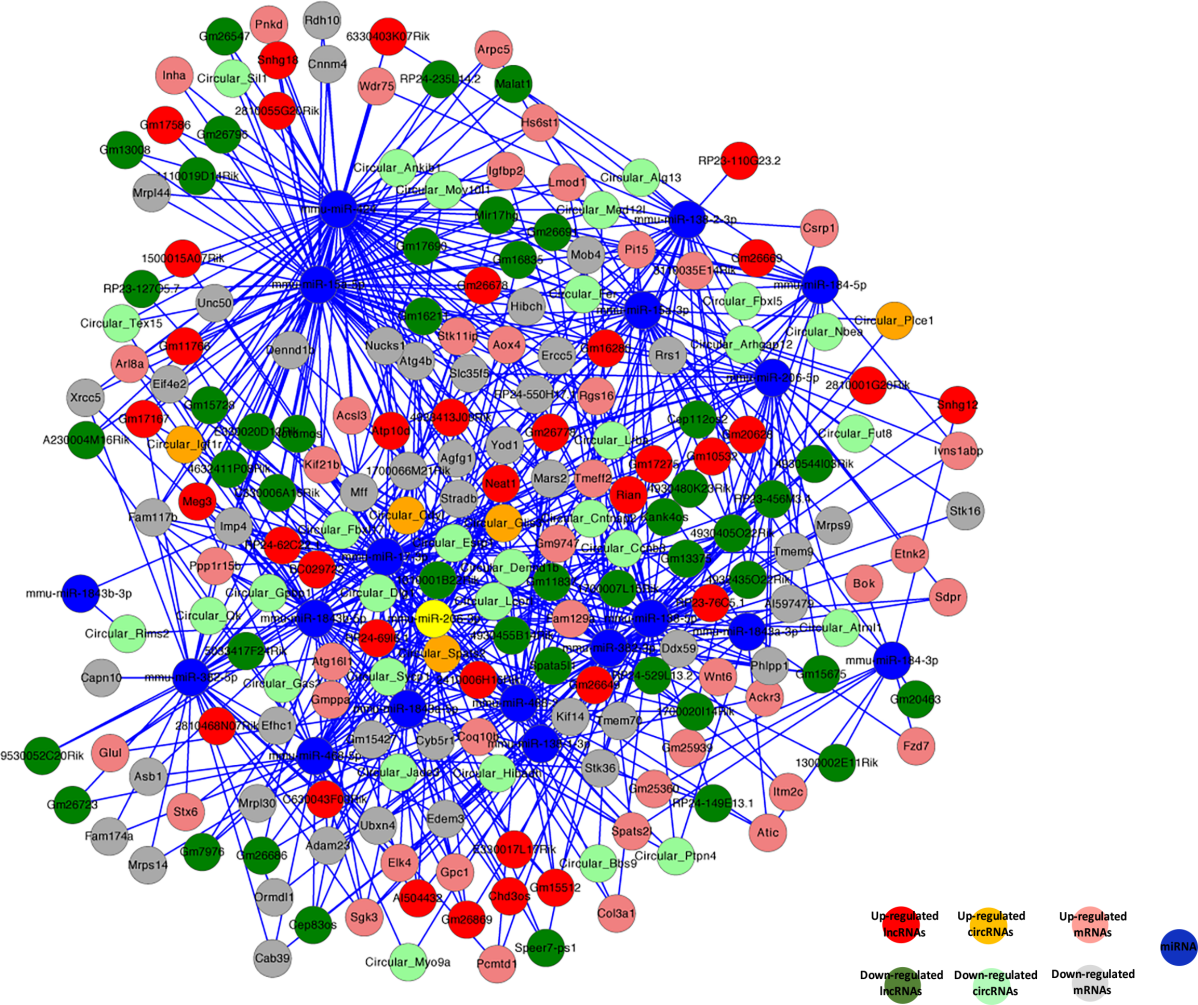
Competing endogenous RNA network in germline stem cells The competing endogenous RNA network is based on lncRNA/miRNA, circRNA/miRNA, and miRNA/mRNA interactions. The edges represent sequence matching, and lncRNAs or circRNAs connect expression correlated mRNAs via miRNAs.

Recent studies have shown that RNAs regulate each other with microRNA (miRNA) response elements (MREs) though a mechanism named the “competing endogenous RNA (ceRNA)” hypothesis. MREs implicated in ceRNA networks were found to regulate mRNA expression [78]. Accordingly, we constructed a ceRNA network by integrating the expression profiles and regulatory relationships of the mRNAs, lncRNAs, circRNAs, and miRNAs from our high-throughput sequencing data (Figure 9). We selected sex-biased expressed 60 lncRNAs and 29 circRNAs, sharing a common binding site of MRE. For instance, lncRNA Meg3 and cirRNA Igf1r were predicted to be ceRNAs of the miRNA miR-15a-5p, which targets the Inha, Acsl3, Kif21b, and Igfbp2 mRNAs. These sex-biased expressed lncRNAs and circRNAs were also implicated in a number of biological processes, including reproduction, regulation of stem cell differentiation, reproductive process, regulation of genetic imprinting, genetic imprinting, cell differentiation, sex differentiation, regulation of gene expression by genetic imprinting, and sex chromatin. The ceRNA regulatory networks, which include mRNAs, miRNAs, lncRNAs, and circRNAs, might act a pivotal part in germline stem cell acquisition of the sex-specific properties required for differentiation into gametes.

## DISCUSSION

Formation and development of sexually dimorphic germ cells are required for the continuation of mammalian species. Germline stem cells are pivotal for passing genetic information from generation to generation. The conventional view of gene regulation focused on protein-coding genes, but this changed after the discovery of numerous noncoding RNAs, including lncRNAs and circRNAs. Studies of lncRNA and circRNA expression have revealed their potential roles in many kinds of stem cells. However, comprehensive analyses of differentially expressed profiles of lncRNAs and circRNAs in male and female germline stem cells have not been reported until now. To probe the functions of lncRNAs and circRNAs in germline stem cells, here, we explored the genome-wide expression profiles of lncRNAs and circRNAs in three male mouse germline stem cells and three female mouse germline stem cells using high-throughput sequencing.

A total of 24376 lncRNAs (including 18573 novel lncRNAs) and 18822 circRNAs were identified in the germline stem cells. Six possible novel lncRNAs and six circRNAs were verified using qRT-RCR or RT-RCR. Most of the qRT-RCR or RT-RCR results confirmed the high-throughput sequencing data. Our newly identified lncRNAs in SSCs and FGSCs shared many characteristics. Their lengths were similar to the lengths of known lncRNAs. Further, the novel lncRNAs were shorter, had lower exon numbers, lower expression levels, and were less well conserved than protein-coding transcripts. The lncRNAs were scatted widely on all the chromosomes, and intergenic lncRNAs constituted the majority of the novel lncRNAs. Of the 18822 circRNAs we identified, most were exonic circRNAs, and the expression levels of circRNA hosting genes were significantly higher than the expression levels of other genes.

The mRNAs, lncRNAs and circRNAs expression profiles of the SSCs and FGSCs revealed similar expression patterns and FGSCs response to GDNF signaling in the way similar to SSCs. In addition, 2331 lncRNAs and 921 circRNAs exhibited sex-biased expression between the male and female germline stem cells. The different expression between sex suggested that these lncRNAs and circRNAs may act a pivotal part in reproduction processes including spermatogenesis and oogenesis. Subsequent function analyses of these sex-biased lncRNAs and circRNAs will be help to better understand the spermatogenesis, oogenesis and the sexual fate decision of germ cells in mammals.

What is more, the sex-biased lncRNAs and mRNAs were nonrandomly distributed between the X chromosome and autosomes. Male-biased lncRNAs and mRNAs were enriched on autosomes however female-biased lncRNAs and mRNAs were significantly overrepresented on the X chromosomes. These patterns could be explained by evolutionary models that invoke selection to, a good illustration of this, localize male-beneficial genes off a precociously silenced X during meiosis in male germline stem cell or localize female-beneficial genes on the relatively more-abundant X during meiosis in female gremlin stem cell [66].

GO analysis for the sex-biased lncRNAs and circRNAs showed that some terms under the biological process and molecular function categories were related to the sex-specific properties required for differentiation into SSCs and FGSCs. What is more, KEGG pathway analysis for the sex-biased lncRNAs and circRNAs revealed a large number of pathways that could act pivotal roles in the sex-specific properties of SSCs and FGSCs.

To date, the functions of most lncRNAs are not well understood. Constructing a CNC network allowed the prediction of lncRNAs functions [79]. Our results showed that hundreds of lncRNAs were significantly correlated with dozens of mRNAs. Therefore, the CNC network could be used to further seek the relation of lncRNAs and mRNAs.

Recently, circRNAs were proposed to harbor miRNAs, and were found to be enriched with functional miRNA binding sites. To date, there has been no report on ceRNAs in germline stem cells. Here, for the first time, we constructed a lncRNA–miRNA–circRNA–mRNA ceRNA network for germline stem cells based on our high-throughput sequencing data. Sixty lncRNAs (e.g., Meg3, Atp10b, Rian, Malat1), 29 circRNAs (e.g., Circular_Igf1r, Circular_Gas2, Circular_Cdy, Circular_Ccnb3), and 16 miRNAs (e.g., mmu-miR-424, mmu-miR-15a-5p, mmu-miR-138-5p, mmu-miR-15a-3p) have been included in the ceRNA network. For instance, lncRNA Meg3 and cirRNA Igf1r could bind competitively with miRNA-15a-5p increasing target gene Inha, Acsl3, Kif21b, and Igfbp2 expressions. Our results will help to enrich our understanding of germline stem cells. Further research on ceRNAs of miRNA-15a-3p and other associated functions are being carried out in our laboratory.

Sex determination in germ cells was an extremely important biological event. However, we known little about the mechanism of sex determination in germ cells. Previous study showed that Foxl3 could repress female germline stem cells to enter spermatogenesis, indicating that Foxl3 acted a key germ cell intrinsic factor in sex determination of germ cells in the teleost fish, and medaka [80, 81]. Although Foxl3 was detected in the majority of vertebrate genomes, we did not found Foxl3 in mammalian genomes [81]. This indicated that the sex determination of germ cells in mammals was in a distinct manner in comparison with Foxl3-possessing vertebrates. Our research might help to find the mechanism of the sex determination of germ cells in mammals.

The paternal and maternal imprinting patterns are built during spermatogenesis and oogenesis respectively [82]. Previous study has shown that cultured SSCs possess the potential to be reprogrammed into oocyte-like cells, and the reprogramming from cultured SSCs to oocyte-like cells was accompanied by imprinting reversal [82]. Our study revealed a great deal of lncRNA and circRNA involved in the different genetic imprinting between male and female germline stem cells. Therefore, our research provided a useful research basis to learn epigenetic regulation in gametogenesis and sex reversal.

To conclude, we have described lncRNAs and circRNAs expression profiles that might influence the sex-specific properties required for differentiation into gametes between SSCs and FGSCs in mouse. The results provide a foundation for further research into the functions and mechanisms of lncRNAs and circRNAs in germline stem cells.

## MATERIALS AND METHODS

### Animals

All procedures involving animals were approved by the Institutional Animal Care and Use Committee of Shanghai, and were conducted in accordance with the National Research Council Guide for Care and Use of Laboratory Animals. Ddx4-Cre mice (FVB-Tg(Ddx4-Cre)1Dcas/J) [83] and mT/mG mice (B6.129(Cg)-Gt(Rosa)26Sortm4(ACTB-tdTomato,2EGFP) Luo/J) [84] were purchased from the Model Animal Research Center of Nanjing University, China, and bred according to the instructions from the Jackson Laboratory (Bar Harbor, Maine). Briefly, male Ddx4-Cre mice were bred with wild-type females, and homozygous mT/mG mice were bred together. The mT/mG mice harbor genes for two cell membrane-targeted fluorescent proteins at the Rosa26 locus, namely tdTomato and EGFP. The membrane-targeted tdTomato (mT) cassette with loxP sites on both sides expresses strong red fluorescence in all tissues. When crossed with CRE-expressing mice, the tdTomato cassette is removed by CRE-mediated recombination and the immediately downstream membrane-targeted EGFP (mG) cassette is expressed in the CRE-expressing cells of the offspring [84]. To produce the Ddx4-Cre; mT/mG mice, male Ddx4-Cre mice younger than 63 days old were crossed with female mT/mG mice. The Ddx4-driven CRE was expressed in the germline lineage, which resulted in a change of expression from tdTomato to EGFP in the germ cells of this strain.

### Isolation and purification of FGSCs from mouse ovaries

Ovaries from a total of 500 female mice (Ddx4-Cre; mT/mG mice, aged 3–5 days) were collected, washed with ice-cold phosphate-buffered saline (PBS), and cut into small pieces. Two-step enzymatic isolation of FGSCs was performed, as described previously [12]. Briefly, the mouse ovarian tissue was treated with 1 mg/ml collagenase (Type IV; Sigma), followed by 0.05% trypsin and 1 mM EDTA digestion at 37°C to dissociate cells. After passing through a 13-μm nylon cell filter, the cells were suspended in PBS and subjected to fluorescence-activated cell sorting (FACS), according to the manufacturer's instructions (Beckman Coulter), to sort GFP-positive cells. Then, GFP-positive cells were suspended in PBS and plated in 35 mm cell culture plates precoated with mouse laminin (4. 4 μg/cm^2^). After incubated for 45min at 37°C, unbound cells were removed from bound cells by pipetting.

### Isolation and purification of SSCs from mouse testis

Testis from a total of 50 male mice (Ddx4-Cre; mT/mG mice, aged 6 days) were collected, washed with PBS, and cut into small pieces. Two-step enzymatic isolation of SSCs was performed, as described previously [10]. Briefly, the mouse testis tissue was treated with 1 mg/ml collagenase (Type IV; Sigma), followed by 0.05% trypsin and 1 mM EDTA digestion at 37°C to dissociate cells. After passing through a 13-μm nylon cell filter, the cells were suspended in PBS and subjected to FACS, according to the manufacturer's instructions, to sort GFP-positive cells. Then, GFP-positive cells were suspended in PBS and plated in 35 mm cell culture plates precoated with mouse laminin (4. 4 μg/cm2). After incubated for 45min at 37°C, unbound cells were removed from bound cells by pipetting.

### RT-PCR

Total RNA from germline stem cells were extracted using the Trizol reagent (Qiagen), according to the manufacture's instruction. Reverse transcription was performed using a HiScript^®^IIQRT SuperMix (+gDNA wiper) kit (Vazyme, R223-01), according to the manufacturer's instruction. For RT-PCR, 30 cycles were performed using Taq polymerase (Takara, R10T1M) with primer sets specific for each gene (Supplementary Table 8). Samples were detected using ethidium bromide (EB) staining. PCR products were isolated, sub-cloned, and sequenced to confirm the gene sequences.

### Immunofluorescence staining

The cells in 48 plates were washed with 1× -concentrated phosphate-buffered saline (PBS), fixed in 4% paraformaldehyde (PFA) for 20 min at room temperature, washed twice with PBS, and incubated for 10 min at 37°C in blocking buffer (PBS containing 10% normal goat serum). Then, they were incubated overnight in a humidified chamber at 4°C with one of the following: 1:500 dilution of a rabbit polyclonal anti-Mvh antibody (Abcam); 1:150 dilution of rabbit polyclonal anti-Oct4 (Santa Cruz Biotechnology); 1:200 dilution of a rabbit polcyclonal anti-Dazl antibody (Abcam); 1:100 dilution of rabbit polyclonal anti-GFRA1 (ABclonal). After washing twice with PBS, the cells were incubated at 37°C for 30 min with a 1:150 dilution of tetramethylrhodamine isothiocyanate (TRITC) conjugated secondary antibody (goat anti-rabbit IgG, then incubated at 37°C for 20 min with 500 ng/mL 4′,6-diamidino-2-phenylindole (DAPI; Sigma). The cells were subsequently mounted in anti-fade mounting medium. Images were obtained using a Leica DMI3000 B microscope and a Leica DFC550 digital camera.

### Cell proliferation assay

SSCs and FGSCs were incubated in SSC culture medium [11] and FGSC culture medium [12], respectively, for two days. After that, the cells were incubated in culture medium that contained 10 mM EdU (Invitrogen Life Sciences) for 3 h at 37°C. EdU is a nucleoside analog of thymidine that is incorporated into DNA during active DNA synthesis [85]. EdU staining was performed with a Click-iT^®^ Plus Edu Alexa Fluor^®^ 555 Imaging kit (Invitrogen Life Sciences) according to the manufacturer's instructions. In brief, cells were fixed with 3.7% paraformaldehyde for 15 min at room temperature, washed twice with 3% bovine serum albumin (BSA), permeated by 0.5% Triton X-100 for 20 min at room temperature, washed twice with 3% BSA, incubated with the Click- iT^®^ Plus reaction cocktail for 30 min at room temperature, and washed once more with 3% BSA.

### RNA extraction and qualification

Total RNA from the SSCs or FGSCs was isolated using TRIzol reagent (Invitrogen, Life Technologies, USA) according to the manufacturer's protocol. Total RNA from each sample was quantified using Agilent 2100, and RNA integrity was assessed using Agilent 2100.

### Library preparation and sequencing

After extracting the total RNA, mRNA, and noncoding RNAs were enriched by removing rRNA from the total RNA with kit (Arraystar rRNA Removal Kit). The mRNAs and noncoding RNAs were fragmented into short fragments (about 200–500 nt) using the fragmentation buffer. First-strand cDNA was synthesized by random hexamer primer using the fragments as templates. During second strand synthesis, dTTP was substituted by dUTP. Short fragments were purified and resolved with EB buffer for end reparation and single nucleotide A (adenine) addition. The purified short fragments were connected with adapters, then the second strand was degraded using UNG (uracil-N-glycosylase) [86]. After agarose gel electrophoresis, the suitable fragments were selected as templates for PCR amplification. An Agilent 2100 Bioanaylzer and ABI StepOnePlus Real-Time PCR System were used for quantification and qualification of the sample library. The library was sequenced using a Illumina HiSeq^TM^ 2000 system.

### Transcript assembly

Reads that mapped to the mouse genome were assembled using Cufflinks [54]. We performed a Reference Annotation-Based Transcript (RABT) [87] assembly with the reference gene annotations to compensate for the incompletely assembled transcripts caused by read coverage gaps in some regions of the reference genes. Faux-reads were generated from reference transcripts in order to capture features in the reference genes that could be missing in the sequencing data due to low coverage; these reads were merged with the (aligned) sequenced reads for assembly. The set of short transcribed fragments generated in the last step was subsequently compared with the reference transcripts to remove any short transcribed fragments that were approximately equivalent to the whole or a portion of a reference transcript.

### LncRNA identification

To reduce false-positive rates, we used the following six steps to identify lncRNAs, including lncRNAs, intronic lncRNAs, and anti-sense lncRNAs [58]: 1) All assembled transcripts of two sequencing libraries were combined using Cuffcompare software [88], and transcripts if they were assembled only by Scripture (beta2) [54] or Cufflinks v2.1.1 [55] were discarded. 2) Transcripts with a single exon and less than 200-bp long were removed. 3) The read coverage of every transcript was calculated using Cufflinks v2.1.1 [55], and transcripts with less than three reads of coverage and an FPKM value less than 0.01 were removed. 4) The remaining transcripts were searched against known lncRNAs in ALDB (domestic animal long noncoding RNA database) [89] using Cuffcompare. Only lncRNA transcripts in which the splice sites were completely congruent between our results and those annotated in ALDB were immediately considered as known lncRNAs. Transcripts that were identified as rRNA, tRNA, snRNA, snoRNA, pre-miRNA, or pseudogenes were discarded. 5) Transcripts that matched known mRNAs (ftp://ftp.ensembl.org/pub/release-79/fasta/sus_scrofa/dna) were also discarded. Three kinds of lncRNAs were identified from the remaining transcripts using the class code information from Cuffcompare (http://cole-trapnell-lab.github.io/cufflinks/cuffcompare/index.html#transfrag-class-codes). 6) Coding-Non-Coding Index (CNCI) (score < 0) [90] and Coding Potential Calculator (CPC) (score < −1) [91] were used to assess the coding potential of the remaining transcripts, and the intersection of the results from both software was used to define novel lncRNA transcripts.

### CircRNAs identification

The Fastq reads obtained after sequencing each sample were first mapped to the mouse mm9 genome by Bowtie [92], allowing 2 mismatches. After removing PCR-duplicated reads with the FASTX toolkit (http://hannonlab.cshl.edu/fastx_toolkit/), all the unmapped reads were aligned using BLAT (no mismatch or gap allowed). Dual alignments of two complimentary segments within a single read mapping to two regions on the same chromosome in the reverse order and no more than 100 kb away from each other were selected as candidate circular-junction transcripts. Next, GT and AG dinucleotides were searched for within 10-nucleotide genomic windows flanking the donor and acceptor ends of each junction, respectively. Candidates with GT/AG flanking junctions were retained, and the GT/AG dinucleotides were used to identify the precise splice sites.

### Expression analysis

The expression levels of the lncRNAs, circRNAs, and protein-coding genes were estimated by FPKM and assessed using Cuffdiff v2.1.1 [93]. FPKMs were computed by summing the FPKMs of the transcripts in each gene group. Cuffdiff provides statistical routines for determining differential expression in digital transcript or gene expression data using a model based on the negative binomial distribution. P-adjust <0.05 and |log_2_(fold change)| >1 were set as the threshold in the differential expression analysis.

### GO and KEGG pathway analysis

We conducted GO analysis (http://www.geneontology.org) to annotate the genes with terms under the biological process, cellular component, and molecular function categories. The log10 (*p*-value) denotes enrichment scores that represent the significance of GO term enrichment among differentially expressed genes. We also performed KEGG pathway analysis to predict the molecular interactions and reaction networks associated with differentially regulated genes. The −log10 (*p*-value) denotes an enrichment score for the significance of the pathway correlations.

### Correlation and co-expression analysis

The co-expression analysis was conducted by calculating the Pearson correlation coefficient (PCC) between coding genes and noncoding transcripts according to their expression levels. The network construction procedures: (i) preprocessed data: the same coding gene with different transcripts of the median value represent the gene expression values, without specific treatment of the lncRNA expression value; (ii) screen data: remove the subset of data according to the lists that show the sex-biased expression of lncRNA and mRNA; (iii) calculate the Pearson correlation coefficient and use the R-value to calculate the correlation coefficient of PCC between the lncRNA and coding genes; and (iv) screen using Pearson correlation coefficient, which was selected when PCC ≥ 0.99 as meaningful and draw the NCN network using cytoscape.

### Competing endogenous RNA (ceRNA) network analysis

The lncRNAs, circRNAs, and mRNAs with expression levels that shared a meaningful correlation were subjected to the ceRNA analysis. Potential miRNA response elements (MREs) were searched for in the lncRNA, circRNA, and mRNA sequences, and overlapping of the same miRNA seed sequence binding site in both the lncRNA/circRNA and mRNA sequences was considered to predict lncRNA/circRNA–miRNA–mRNA interaction. MiRNA binding sites were predicted by miRcode (http://www.mircode.org/), and miRNA–mRNA interactions were predicted by TargetScan (http://www.targetscan.org/).

### Data access

The high-throughput sequencing data for the male and female germline stem cells has been deposited in the Gene Expression Omnibus (GEO) under accession number GSE87824.

## SUPPLEMENTARY MATERIALS FIGURES AND TABLES
















