# Correction: Systematic identification and comparison of expressed profiles of lncRNAs and circRNAs with associated co-expression and ceRNA networks in mouse germline stem cells

**DOI:** 10.18632/oncotarget.25674

**Published:** 2018-06-15

**Authors:** Xiaoyong Li, Junping Ao, Ji Wu

**Affiliations:** ^1^ Renji Hospital, Key Laboratory for the Genetics of Developmental and Neuropsychiatric Disorders (Ministry of Education), Bio-X Institutes, School of Medicine, Shanghai Jiao Tong University, Shanghai, 200240, China; ^2^ State Key Laboratory of Oncogenes and Related Genes, Shanghai Cancer Institute, Renji Hospital, Shanghai Jiao Tong University School of Medicine, Shanghai, 200032, China; ^3^ Key Laboratory of Fertility Preservation and Maintenance of Ministry of Education, Ningxia Medical University, Yinchuan, 750004, China; ^4^ Shanghai Key Laboratory of Reproduction Medicine, Shanghai, 200025, China

**This article has been corrected:** The correct figure 4 (D, E) is given below:

The authors declare that this correction does not change the results and conclusions of this paper.

**Figure 4:**
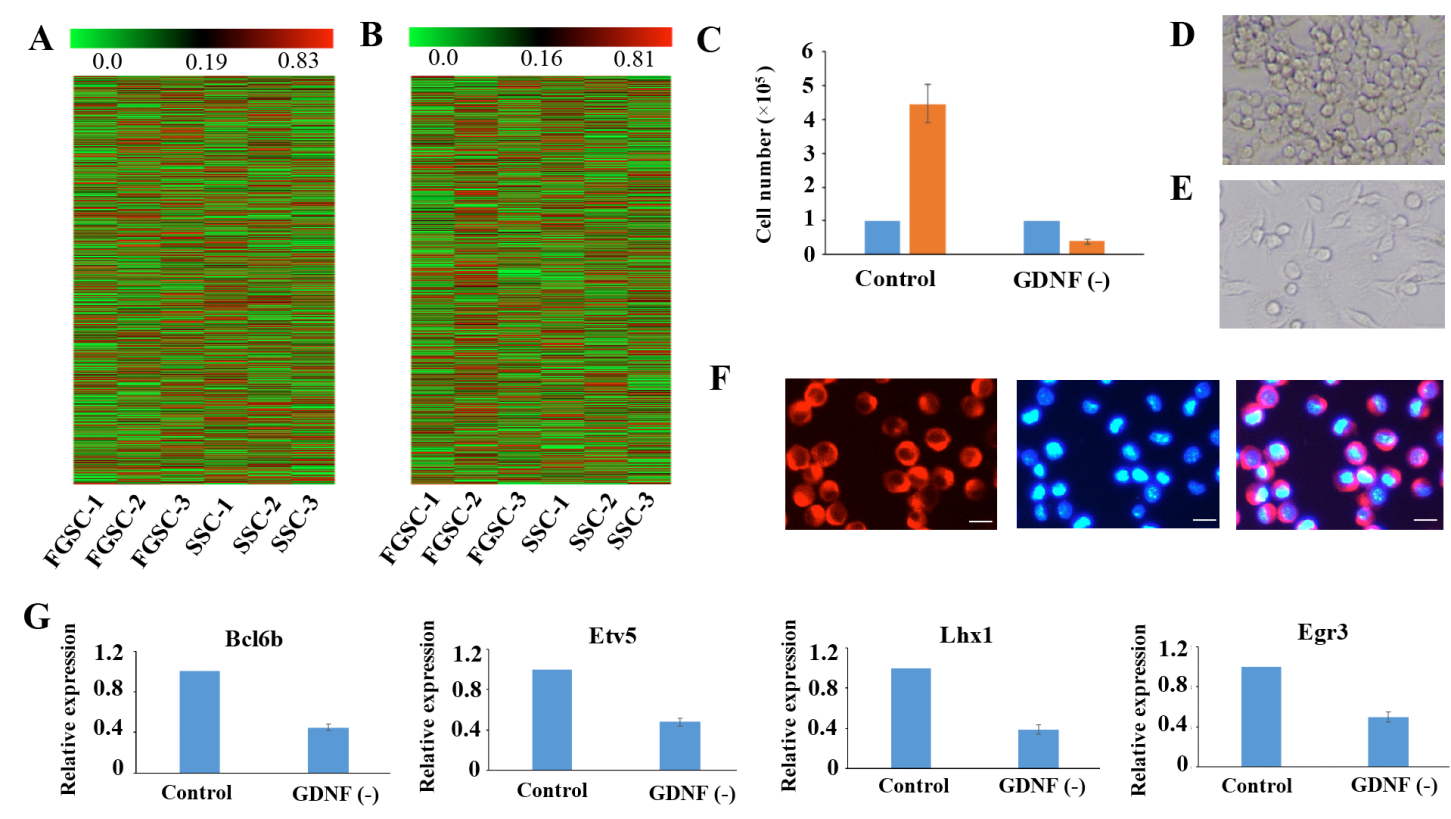
Similar lncRNA profiles and self-renewal mechanisms shared by mouse SSCs and FGSCs (**A**–**B**). Heat map showing expression profiles of mRNAs (A) and lncRNAs (B). The maps are based on the expression values of all expressed lncRNAs and mRNAs detected by high-throughput sequencing. The color scale indicates the expression values; the intensity increases from green to red. Each column represents one sample, and each row represents a transcript. (**C**–**E**) Proliferation of FGSCs in culture was dependent on the glial cell line-derived neurotrophic factor GDNF. Cells plated at 1.0 × 105 per well into culture medium without GDNF did not proliferate (average 0.4 × 105 per well) after a week. FGSCs were cultured with culture medium containing GDNF as the control. Error bars indicate the standard deviation (SD). Each experiment was conducted three times. (**F**) The FGSCs was detected by immunofluorescence analysis with the antibodies against GFRA1. Left, GFRA1 immunofluorescence. Middle, DAPI immunofluorescence. Right, merge for GFRA1 and DAPI immunofluorescence Scale bars: 10 μm. (**G**) Withdrawal of GDNF for a week resulted in a significant change in the expression levels of self-renewal-related genes that showed the largest response to the GDNF signal, including Bcl6b, Etv5, Lhx1, and Egr3. GAPDH was used as an internal control. Error bars indicate the standard deviation (SD). Each experiment was conducted three times.

Original article: Oncotarget. 2017; 8:26573-26590. https://doi.org/10.18632/oncotarget.15719

