# Supplementary material for: Systematic identification and comparison of expressed profiles of lncRNAs and circRNAs with associated co-expression and ceRNA networks in mouse germline stem cells
Source: Oncotarget. 2017 Feb 25;8(16):26573–90. doi: 10.18632/oncotarget.15719 (PMC5432280; doi:10.18632/oncotarget.15719)
Supplement: Supplementary file 1 [file oncotarget-08-26573-s001.pdf]

Systematic identification and comparison of expressed profiles of lncRNAs and circRNAs with associated co-expression and ceRNA networks in mouse germline stem cells

Supplementary Materials

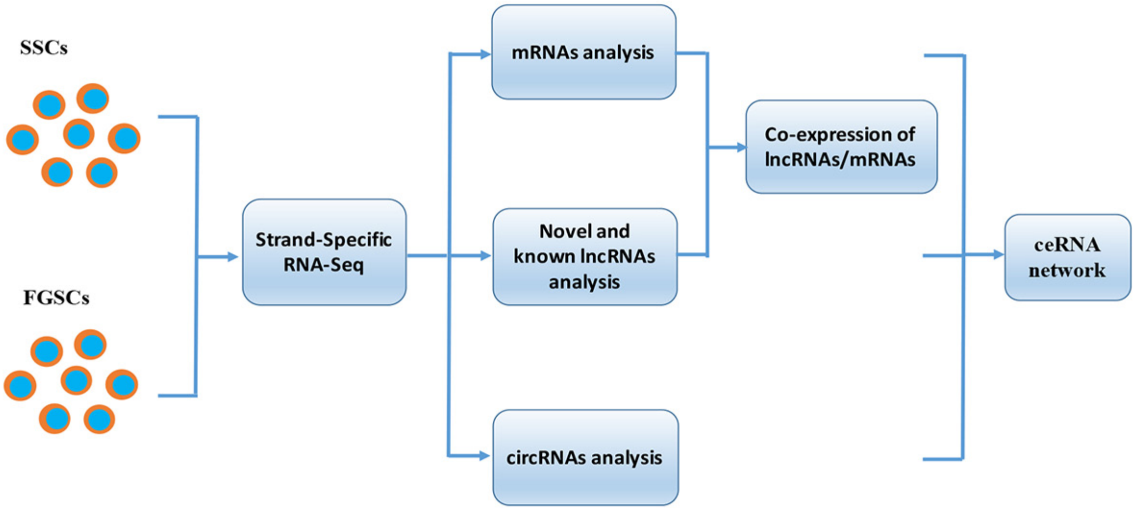

Supplementary Figure 1: Design and overview of the experimental approaches used in this study.

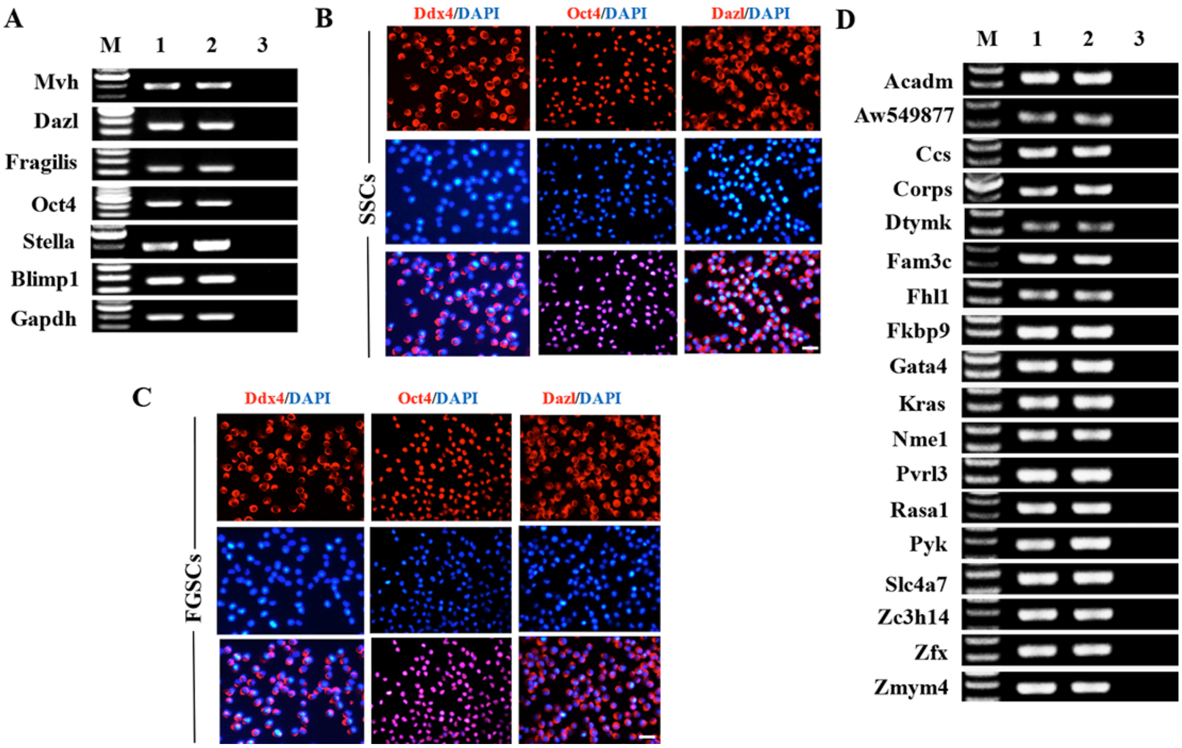

Supplementary Figure 2: Characterization of mouse SSCs and FGSCs. (**A**) RT-RCR analysis of germ cell markers in SSCs and FGSCs. M, 100bp DNA maker; lane 1, SSCs; lane 2, FGSCs; lane 3, no template control. (**B**) The SSCs was detected by immunofluorescence analysis with the antibodies against Mvh, Oct4 and Dazl. Scale bars: 20 μm. (**C**) The SSCs was detected by immunofluorescence analysis with the antibodies against Mvh, Oct4 and Dazl. Scale bars: 20 μm. (**D**) Eighteen core stemness genes detected by our previous study are expression in both SSCs and FGSCs. M, 100bp DNA maker; lane 1, SSCs; lane 2, FGSCs; lane 3, no template control.

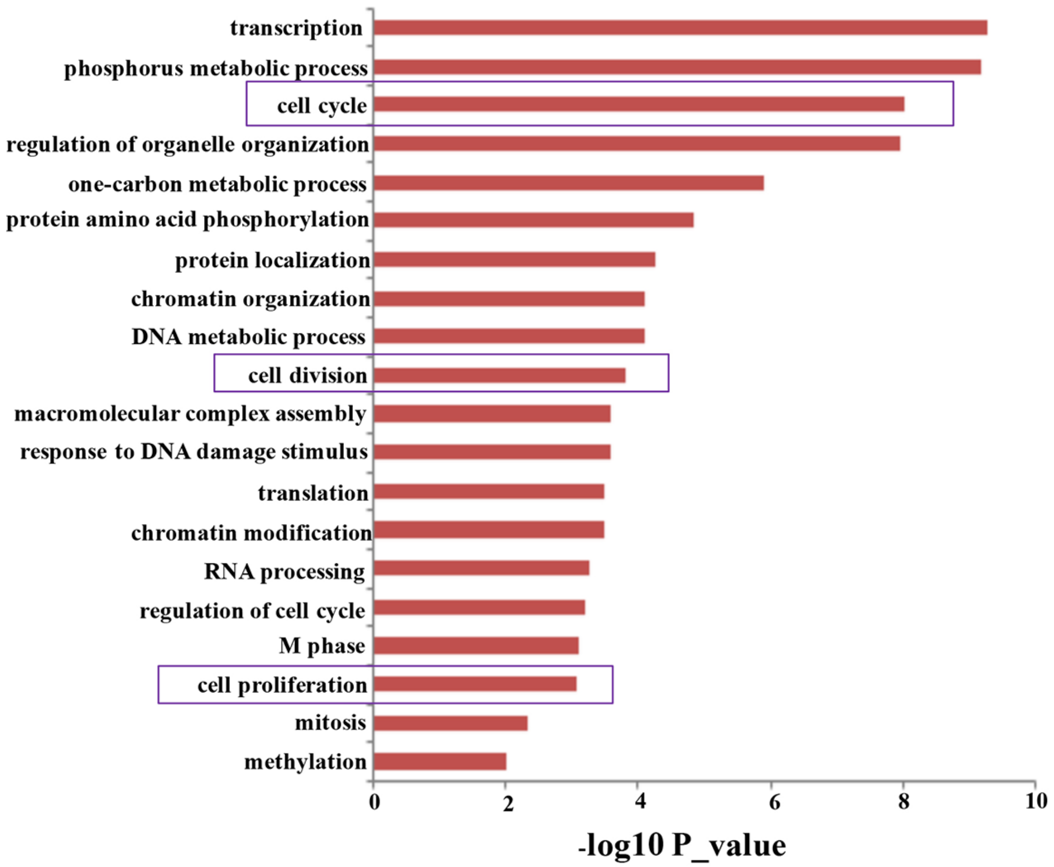

Supplementary Figure 3: Gene Ontology (GO) analyses of co-highly expressed genes. GO annotation of co-highly expressed genes with top 20 Enrichment score covering domains of biological processes.

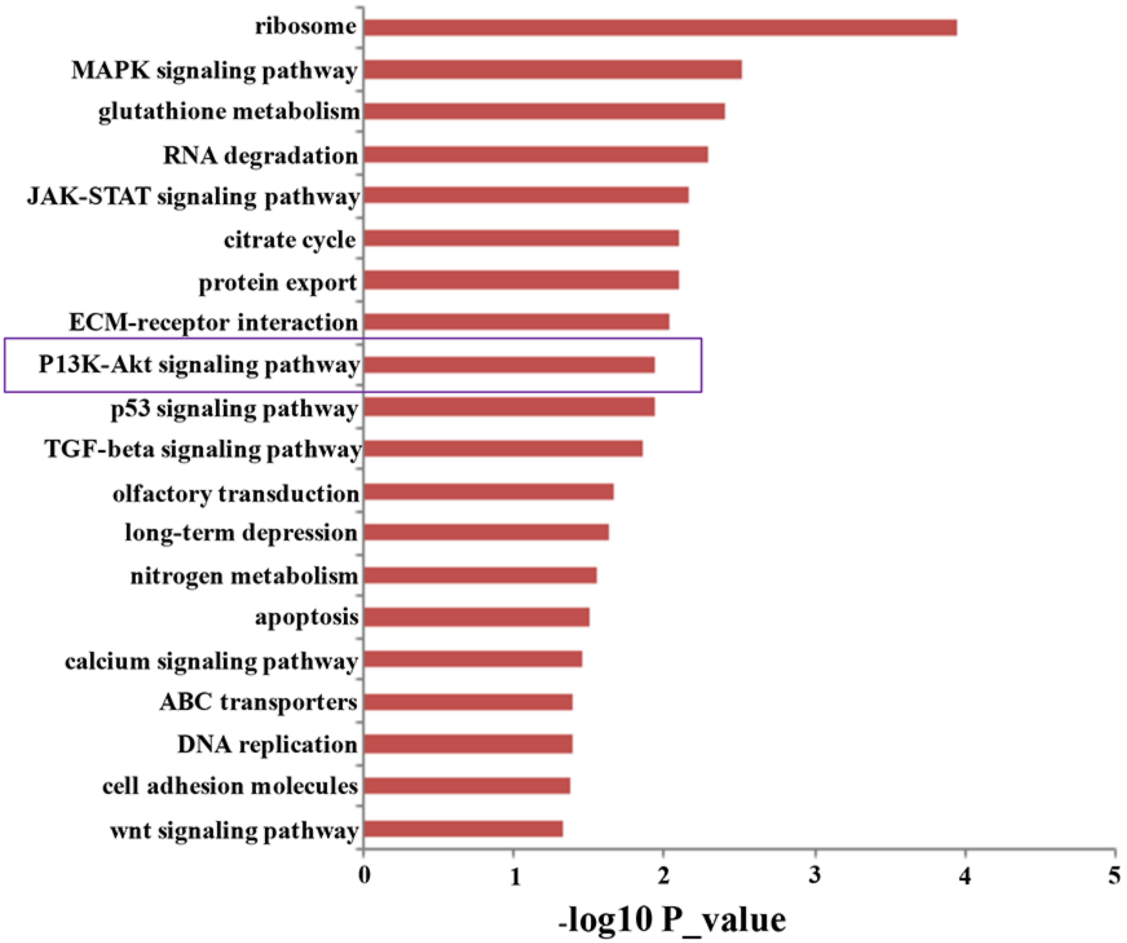

Supplementary Figure 4: KEGG pathway analyses of co-highly expressed genes. KEGG pathway annotation of co-highly expressed genes with top 20 Enrichment score covering domains of biological processes.

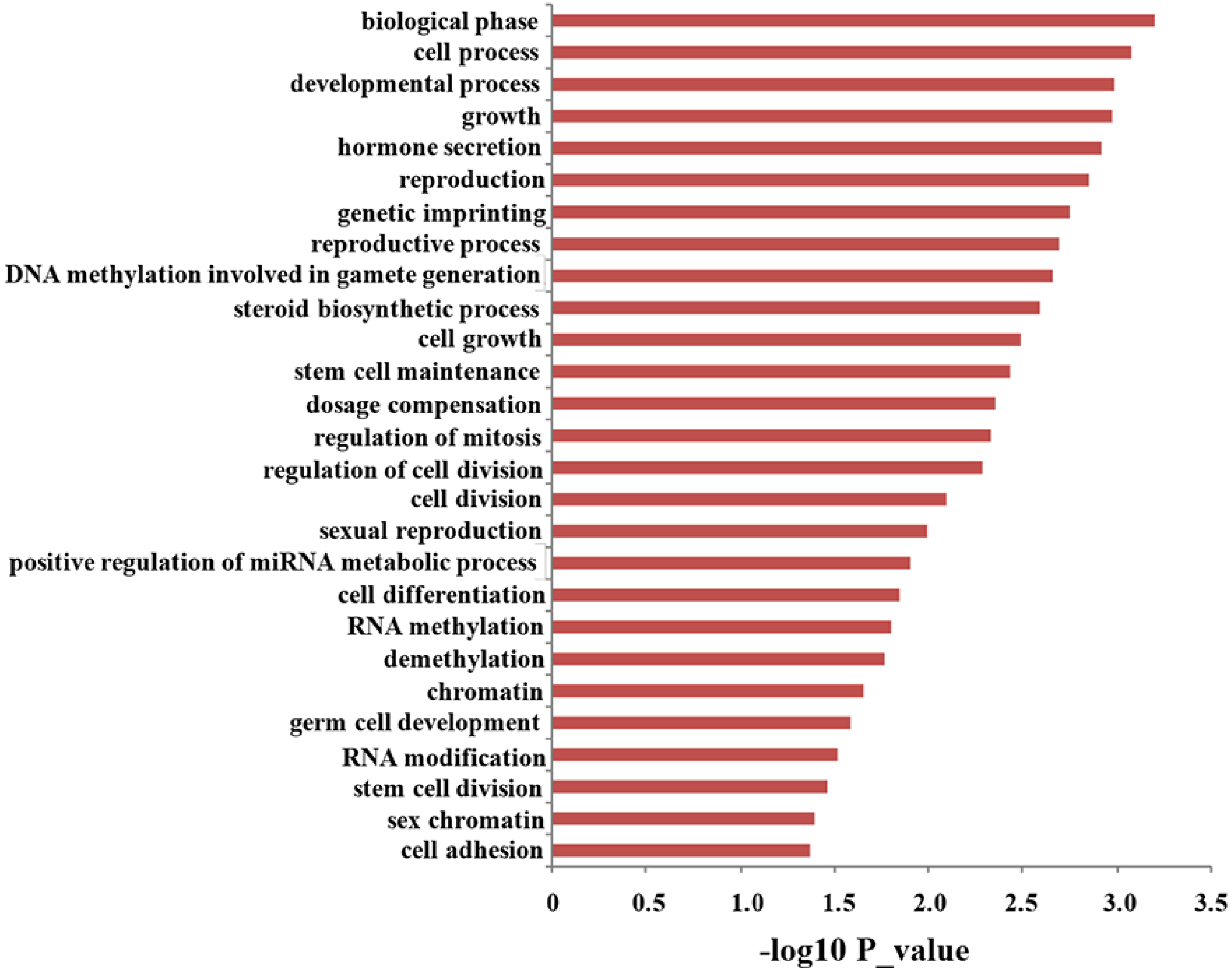

Supplementary Figure 5: Gene Ontology (GO) analyses of circRNA hosting genes. GO annotation of circRNA hosting genes with 27 Enrichment score covering domains of biological processes.

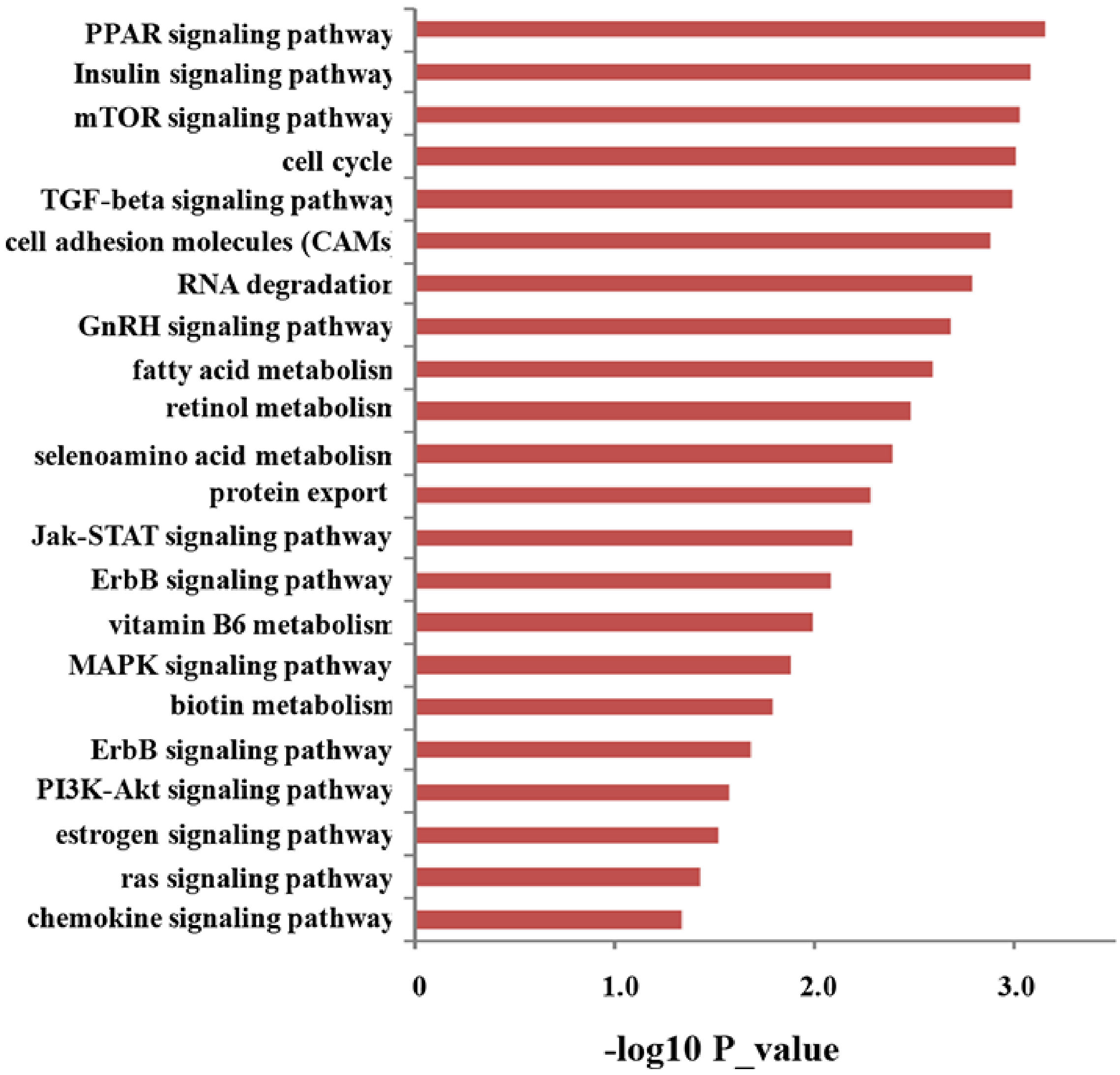

Supplementary Figure 6: KEGG pathway analyses of circRNA hosting genes. KEGG pathway annotation of circRNA hosting genes with 22 Enrichment score covering domains of biological processes.

Supplementary Table 1: Novel LncRNAs identified in both SSCs and FGSCs.
See Supplementary_Table_1

Supplementary Table 2: Known LncRNAs identified in both SSCs and FGSCs.
See Supplementary_Table_2

Supplementary Table 3: Full information lists of GO terms of sex-biased lncRNAs.
See Supplementary_Table_3

Supplementary Table 4: Full information lists of KEGG pathway of sex-biased lncRNAs.
See Supplementary_Table_4

Supplementary Table 5: CircRNAs identified in both SSCs and FGSCs. See Supplementary_Table_5

Supplementary Table 6: Full information lists of GO terms of sex-biased circRNAs. See Supplementary_Table_6

Supplementary Table 7: Full information lists of KEGG pathway of sex-biased circRNAs.
See Supplementary_Table_7

Supplementary Table 8: Primer sequence used in RT-PCR analysis

Gene name	Forward	Reverse	PCR product size
Mvh	GCCAGAGGGCTTGATATTGA	CAACTGGATTGGGAGCTTGT	370
Dazl	AATGACGTGGATGTGCAGAAG	ACAGTTGTATAAGCCTGGTAGTT	320
Fragills	AGCCTATGCCTACTCCGTGA	GGGTGAAGCACTTCAGGACC	225
Oct4	GTTCAGCCAGACCACCATCT	TGGGAAAGGTGTCCCTGTAG	385
Stella	ACCATCAGAGAAAGTCGACCC	AATGGCTCACTGTCCCGTTC	372
Blimp1	GAGTAGTCAGTCGCTCGCTC	TCCAACATCTGAGATAAGCCTCT	241
GAPDH	ACTCAGGAGAGTGTTTCCTCG	GCGGAGATGATGACCCTTTTG	396
Acadm	GGTGACGAGTATGTTATC	TGTAGGTCTGGTTCTATC	312
AW549877	ATCAATTACTTGCTACAG	CATCAGTATCAACTTCAT	214
Ccs	GTAGCCAATTACAGAATC	AGCTGTTTATCCTCTATC	325
Corps	AATCCTTACGATGCTGAT	AAGAACACAACTCTAACAC	393
Dtymk	AACTGGAAGGTGGTTGAT	GCTGAATAGTGTAGAGATGTG	366
Fam3c	AAGAATAATGTCGGAAGA	TTATGTGCTGTTCAAAG	314
Fhl1	CTTCTACTGTGTGACTTG	AAAGTTCTTGTAGCAATC	208
Fkbp9	CTTTGACACATACATTGG	ATTGTAGTGGTATTTGAG	280
Gsta4	AGGAGAGAGTCAGGATTG	ACTTCTTAATTGTAGGAATGTTG	330
Kras	GACATTAACCTCCCAGCCGT	GAGAGACAGGCTGAAAGCCA	209
Nme1	CCTTGTTGGTCTGAAGTT	AGAAGTCTCCTCGTATGG	224
Pvrl3	CCTCAGTATGGATTCTCT	GGTTCAACTAACACAGTC	191
Rasa1	CAATCATGTGAGTTAAGT	CTAATGTCAGTGTTCTTG	322
Ryk	CAAATAAAGAGAAGCAAACATT	AAATTGCCTGTGGATTATTG	249
Slc4a7	CTAACACGAGCCCTGAAA	CAACAAATATCTGACGCTACTT	230
Zc3h14	GTAACAAAGACAACTAACTA	ATAGTAATCTTGACTCATCT	267
Zmym4	TGAAGGAAGATATTCTGT	ATGTAACCATTAGGAAGT	256
Zfx	AAGATGACTTAGGTGGAA	CTCTCCAACAATCACTTC	380
